# Supplementary material for: ACE Phenotyping in Human Blood and Tissues: Revelation of ACE Outliers and Sex Differences in ACE Sialylation
Source: Biomedicines. 2024 Apr 23;12(5):940. doi: 10.3390/biomedicines12050940 (PMC11117852; doi:10.3390/biomedicines12050940)
Supplement: Supplementary file 1 [file biomedicines-12-00940-s001.zip › biomedicines-2930173-supplementary.pdf]

SUPPLEMENTARY MATERIALS.

ACE phenotyping in human blood and tissues: Revelation of ACE outliers and sex differences in ACE sialylation

Enikő Edit Enyedi[1,2], Pavel A. Petukhov[3], Alexander J. Kozuch[4], Steven M. Dudek[4], Attila Toth [1], Miklós Fagyas[1,*] and Sergei M. Danilov[4,*]

[1]Division of Clinical Physiology, Department of Cardiology, University of Debrecen, 22 Moricz Zs, 4032 Debrecen, Hungary
[2]Kálmán Laki Doctoral School of Biomedical and Clinical Sciences, University of Debrecen, Hungary.
[3]Department of Pharmaceutical Sciences, College of Pharmacy, University of Illinois at Chicago, 833 S Wood St, Chicago, IL 60612, USA
[4]Department of Medicine, Division of Pulmonary, Critical Care, Sleep and Allergy, University of Illinois at Chicago, CSB 915, MC 719, 840 S. Wood Ave., Chicago, IL 60612, USA
[*] Correspondence: authors: danilov@uic.edu; +1 (708)-642-0635, fagyasmiklos@med.unideb.hu

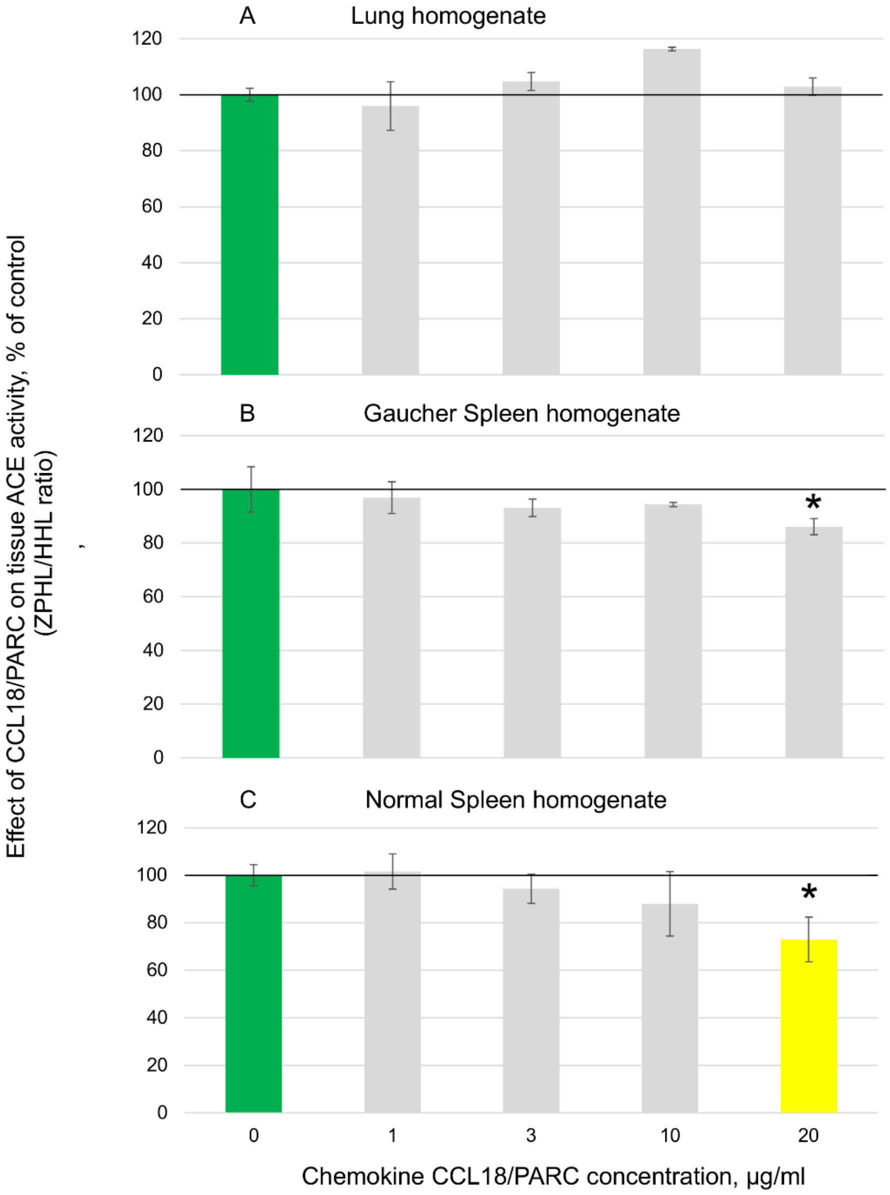

Figure S1. Effect of chemokine CCL18 on catalytic properties of human tissue ACEs.

A-C. Lung (A) and spleen (B-C) homogenates from patient with Gaucher's disease (B) and normal spleen (C) were titrated with different concentration of CCL18. After equilibration, ACE activity was determined with fluorometric assay with two substrates (ZPHL and HHL). Data expressed as a % of ZPHL/HHL ratio from control (without CCL18).

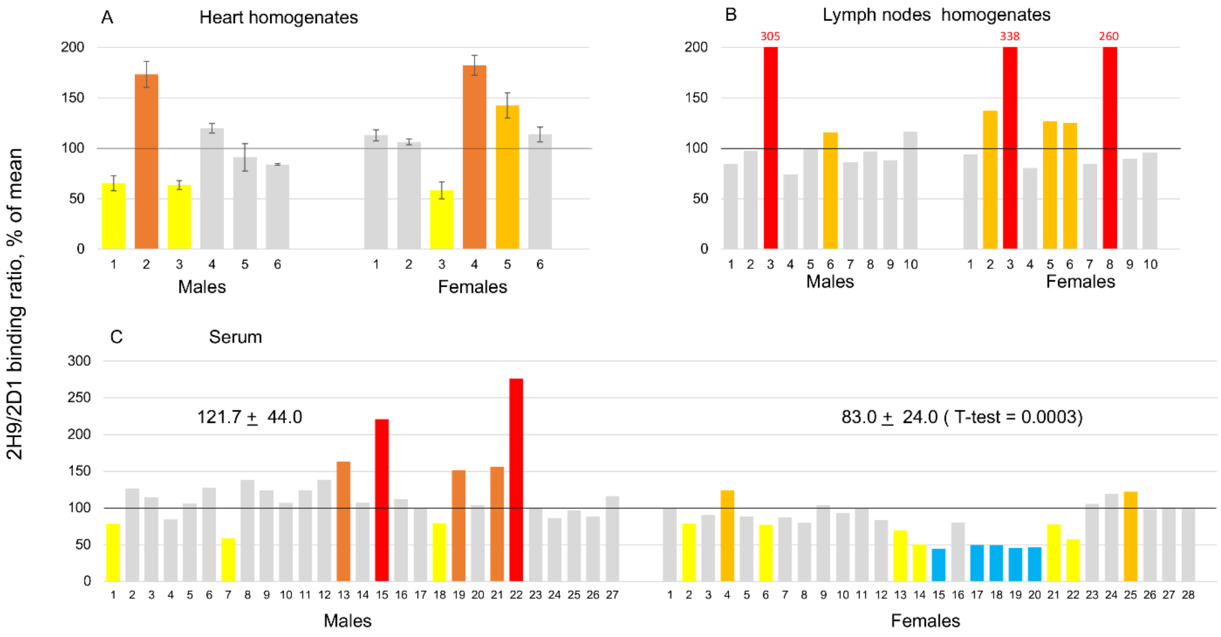

Fig. S2. Gender-specific precipitation of ACE activity from male and female sera samples. ACE activity was precipitated from heart homogenates of 6 males and 6 females (**A**), from lymph nodes homogenates of 10 males and 10 females (**B**) and from 27 male and 28 female sera samples (**C**) by mAbs 2D1 and 2H9, having epitopes on the N and C domains, respectively [Popova, 2021, Kozuch, 2022]. 2H9/2D1 ratios were expressed as % from mean of each tissue set. Mean values from 2-3 experiments (each made in triplicates) ± SD. Red bars on Fig.S2 are represent homogenates of lymph node from unrelated patients (with very low ACE activity), whereas others -from patients with sarcoidosis (and relatively high ACE activity).

Table S1. **Whole exome sequencing analysis of patient S13 (LM1).**

**Parameter**	Index Patient **S13**
## altered genes	11962
## genetic mutations	214698
**Mutations by Type**	
nonsynonymous	10722
frameshifting	246
stop-loss	12
stop-gain	103
synonymous	11528
inframe	392
in noncoding RNA	1805
unknown SNP	277
Unknown InDel	21
damaging variants	1382
ACE mutations	0

Table S2. **All damaging mutations in the genes of Index Patient S13** (Attached Below)

Table S3. **List of 17 patients with WES, which ACE phenotype was determined.**

#	Patient ID	Phenotype	Origin	Year	ACE Mutations	Variants in other genes	
						SNP	Stop/Indels
	Index Patient **S13**	ACE outlier	Debrecen	2022	None	1381	742
Candidates for subtractions							
1	**90B** [14]	Low ACE	Moscow	2019	**Y215C** rs3730025	1388	617
2	**23D** [14]	Low ACE	Moscow	2019	**Y215C** rs3730025	1385	591
3	**47S** [14]	Low ACE	Moscow	2019	None	1378	614
4	**IP1** [13]	High ACE	Albany	2020	**N1196K** rs1033103629	1912	401
5	**IP2** [13]	High ACE	Albany	2020	**P1199L** rs121912703	1245	276
6	**IP3** [13]	High ACE	Moscow	2020	**Q1224X** rs1174820268	1326	314
7	**1AK** [16]	Healthy control	Chicago	2022	None	1323	633
8	**9QN** [16]	ACE outlier	Chicago	2022	None	1435	649
9	**BC107**	Low ACE	Albany	2022	None	1358	580
10	**BC201**	Low ACE	Albany	2022	None	1375	635
11	**S3**	Low ACE	Debrecen	2022	None	1232	566
12	**S15**	Low ACE	Debrecen	2022	None	1181	402
13	**S28**	Low ACE	Debrecen	2022	None	1223	441
14	**S53**	Low ACE	Debrecen	2022	None	1294	463
15	**S63**	Low ACE	Debrecen	2022	None	1278	435
16	**S136**	Low ACE	Debrecen	2022	**Y776X** rs761458810	1295	450

Patients with genomic DNA were subjected to WES. Only mutations with probably and possibly damaging mutations (according to PolyPhen-2 HVAR score) were included. Blue-low blood ACE (< 50% of mean value). Red-high blood ACE (>400% of mean value).

Table S4. **Damaging mutations, unique for Index Patient S13** (Attached Below)

Table S2. All damaging mutations in the genes of Index Patient S13

Genomic DNA from Patient S13 was subjected to WES. Included are damaging mutations: 103 stopgain mutations, 641 nonsense/indels mutations, 12 stoploss mutations, and 1381 damaging missense mutations arranged according to ascending PolyPhen-2, HVAR scores. There are two prediction classifications **D** (Probably damaging, score>=0.909), **P** (**possibly** damaging, **0.446**<=score<=**0.908**). Mutations of interest are highlighted in yellow – ABO (rs8176743), ADAMTSL4 (rs199599791), NOD2 (rs2066844), SAA1 (rs1136747), and SMPD1 (rs550365194).

NO. SNP	GeneName	AAChange	ID	PolyPhen-2 (HVAR)	Genotype
1	APOL3	p.S39R	rs132653	0.447,P	T/T
2	OR4A47	p.V145M	rs7103992	0.447,P	A/A
3	ABCA6	p.N1322S	rs2302134	0.450,P	T/C
4	TAF1B	p.T487M	rs16867245	0.451,P	C/T
5	HAP1	p.S58T	rs4796603	0.451,P	T/T
6	PPP1R16B	p.K473Q	rs61752055	0.451,P	A/C
7	TAS2R31	p.L98P	rs73049067	0.451,P	A/G
8	PLIN4	p.A1124T	rs7251858	0.452,P	C/T
9	ADAM15	p.P769S	rs41264285	0.453,P	C/T
10	SLC38A10	p.E676D	rs55872261	0.453,P	C/A
11	RPAP1	p.E506K	rs1200345	0.454,P	C/T
12	PLA2G3	p.S322R	rs2072193	0.454,P	G/C
13	ERCC5,BIVM-ERCC5	p.G1507R	rs9514066	0.454,P	C/C
14	MEOX1	p.S27L	rs9898682	0.454,P	G/A
15	ZNF813	p.Y439F	rs10422163	0.455,P	A/T
16	ZNF664	p.V58G	rs80197353	0.455,P	T/G
17	PSG9	p.A212S	rs150423600	0.457,P	C/A
18	FBXW8	p.R192Q	rs4076700	0.457,P	A/A
19	SPATA31E1	p.P503S	rs75035814	0.457,P	C/T
20	NAA25	p.L915I	rs12298022	0.458,P	G/T
21	ADAMDEC1	p.N365S	rs3765124	0.458,P	A/G
22	HAP1	p.S357L	rs4796693	0.458,P	A/A
23	FAM160B2	p.L684F	rs117802113	0.459,P	C/T
24	SYNPO2L	p.S833Y	rs34163229	0.459,P	G/T
25	HLX	p.Q125H	rs62621984	0.459,P	A/C
26	C5	p.V145I	rs17216529	0.461,P	C/T
27	PCDHB6	p.T449I	rs17844439	0.461,P	C/T
28	LYZL1	p.K13R	rs2532753	0.461,P	G/G
29	SLC24A1	p.T37S	rs3743171	0.461,P	A/T
30	TBKBP1	p.A508T	rs80267077	0.461,P	G/A
31	HPS4	p.V570M	rs5752330	0.462,P	T/T
32	FEZ2	p.R356C	rs848642	0.462,P	A/A
33	PKD1L2	p.Q120L	rs7191351	0.463,P	A/A
34	CACNA1S	p.L458H	rs12742169	0.466,P	A/T

35	SERPINB11	p.S303P	rs1395267	**0.466,P**	T/C
36	PALM3	p.T412R	rs75389771	**0.466,P**	C/C
37	CCDC122	p.I269T	rs9567280	**0.466,P**	A/G
38	GAGE12J	p.R28Q	rs7064530	**0.467,P**	A/A
39	ZNF607	p.K531R	rs958305	**0.467,P**	C/C
40	IL16	p.P434S	rs4072111	**0.468,P**	C/T
41	KMT2C	p.T316S	rs10454320	**0.469,P**	T/A
42	CMYA5	p.D190G	rs10942901	**0.469,P**	A/G
43	PDIA6	p.K266R	rs4807	**0.469,P**	T/C
44	TBC1D10A	p.R411H	rs4823086	**0.469,P**	C/T
45	ADGRF2	p.I399V	rs9381594	**0.470,P**	A/G
46	B3GLCT	p.E370K	rs1041073	**0.472,P**	G/A
47	INCENP	p.M506T	rs2277283	**0.472,P**	T/C
48	COL16A1	p.T62K	rs2228552	**0.473,P**	G/T
49	ZNF681	p.K479R	rs1852432	**0.474,P**	T/C
50	KIF26B	p.T1302M	rs199742874	**0.475,P**	C/T
51	GPR35	p.T139M	rs3749171	**0.477,P**	C/T
52	CHRNA9	p.A315V	rs55633891	**0.477,P**	T/T
53	HLA-C	p.T118I	rs1131119	**0.479,P**	G/A
54	OR13C5	p.M258T	rs1851724	**0.479,P**	A/G
55	ABO	p.G235S	rs8176743	**0.480,P**	C/T
56	CECR2	p.R293H	rs5747211	**0.482,P**	G/A
57	NOD2	p.R702W	rs2066844	**0.483,P**	C/T
58	CCDC129	p.A42D	rs7811042	**0.483,P**	A/A
59	CD163L1	p.L523M	rs6488268	**0.484,P**	T/T
60	MUC4	p.T3659R	rs779277004	**0.485,P**	G/C
61	KIR3DL1,KIR3DS1	p.G259R	rs1049215	**0.486,P**	G/C
62	NOXO1	p.R39H	rs117304081	**0.486,P**	C/T
63	OR13D1	p.L64V	rs13294411	**0.486,P**	C/G
64	GOLGA6L2	p.K342E	rs35708006	**0.486,P**	C/C
65	SAMD9	p.V549L	rs10279499	**0.489,P**	C/A
66	TPSB2	p.G23V	rs201836020	**0.489,P**	C/A
67	LY6G6D	p.L9V	rs3749952	**0.490,P**	T/G
68	C3orf20	p.L422V	rs6790129	**0.490,P**	C/G
69	ERO1B	p.D129V	rs2477599	**0.491,P**	T/A
70	OR5H15	p.T167S	rs4133322	**0.491,P**	T/T
71	PRSS1	p.N182K	rs1348773645	**0.492,P**	C/G
72	MUC4	p.E953Q	rs13095016	**0.492,P**	G/G
73	SPTBN5	p.R1345H	rs2290559	**0.493,P**	C/T
74	FUT5	p.P187L	rs778970	**0.493,P**	G/A
75	ZAN	p.F1969L	rs542137	**0.495,P**	C/G
76	ITSN2	p.V277I	rs7603997	**0.496,P**	C/T
77	USP29	p.N368S	rs1027392	**0.497,P**	A/G
78	C8orf46	p.A79S	rs61736270	**0.497,P**	G/T
79	OR51I1	p.A252S	rs1498486	**0.500,P**	C/A

80	KRT37	p.P434S	rs17737019	**0.500,P**	G/A
81	NAT2	p.I114T	rs1801280	**0.500,P**	C/C
82	CLIP1	p.T27M	rs34292795	**0.500,P**	G/A
83	SLC26A10	p.A193T	rs923828	**0.501,P**	G/A
84	KRT5	p.G138E	rs11170164	**0.503,P**	C/T
85	C15orf41	p.L73V	rs3784678	**0.503,P**	C/G
86	SYCP2L	p.N647D	rs3798751	**0.503,P**	A/G
87	MUC16	p.S2058P	rs1574479	**0.504,P**	A/G
88	HPS4	p.Q643H	rs1894704	**0.504,P**	A/A
89	CHIA	p.D47N	rs41282494	**0.504,P**	G/A
90	TMEM99	p.I4M	rs17474506	**0.506,P**	C/G
91	MADCAM1	p.P300H	rs3745925	**0.506,P**	C/A
92	OR2T33	p.A169V	rs10888338	**0.507,P**	G/A
93	HSPG2	p.L148L	rs2254357	**0.509,P**	C/G
94	CES1	p.S82L	rs62028647	**0.509,P**	G/A
95	LILRB1	p.R97C	rs766498638	**0.509,P**	C/T
96	PRB4	p.P71T	rs12308381	**0.510,P**	G/T
97	ARSE	p.G449S	rs35143646	**0.511,P**	T/T
98	PRRC2A	p.R1740H	rs1046089	**0.513,P**	G/A
99	OLFM2	p.T127M	rs11556087	**0.513,P**	G/A
100	CTBP2	p.Q539E	rs2946994	**0.513,P**	G/C
101	OR5L1	p.R54W	rs34961497	**0.513,P**	C/T
102	ANKRD18A	p.Y750C	rs2799163	**0.515,P**	T/C
103	KRTAP4-8	p.R126H	rs80168553	**0.515,P**	C/T
104	OR4N2	p.T7R	rs72663752	**0.517,P**	C/G
105	COL6A3	p.K1754R	rs77632596	**0.517,P**	T/C
106	MUC6	p.P1878S	rs200932890	**0.518,P**	G/A
107	CLDN23	p.V210M	rs12548737	**0.521,P**	G/A
108	VPS26A	p.R110H	rs146283722	**0.521,P**	G/A
109	TCF19	p.P241L	rs2073724	**0.521,P**	C/T
110	TAS2R46	p.L228M	rs2708380	**0.521,P**	T/T
111	TAS2R19	p.G282R	rs72475481	**0.521,P**	C/T
112	C8orf44	p.F148S	rs1057463	**0.522,P**	T/C
113	TJP1	p.I794V	rs2229515	**0.522,P**	T/C
114	CDHR3	p.L506F	rs76067797	**0.522,P**	C/T
115	CHST13	p.A271V	rs1056523	**0.523,P**	C/T
116	NBEA	p.I2501V	rs11538677	**0.525,P**	A/G
117	PAK7	p.R335P	rs11700112	**0.525,P**	C/G
118	CLRN2	p.L113V	rs13147559	**0.526,P**	C/G
119	MGME1	p.S15C	rs11551768	**0.527,P**	A/T
120	BOD1	p.V136M	rs72822197	**0.528,P**	C/T
121	FAM131C	p.R245W	rs77667563	**0.528,P**	G/A
122	GEMIN4	p.R1033C	rs7813	**0.528,P**	G/A
123	MKI67	p.T2720P	rs1050767	**0.529,P**	T/G
124	ADAMTSL1	p.E990A	rs41268983	**0.530,P**	A/C

125	RELL2	p.G196R	rs17855844	0.531,P	G/C
126	ZFHX4	p.P1273S	rs61729527	0.531,P	C/T
127	MUC4	p.P1376L	rs78009073	0.531,P	A/A
128	REPIN1	p.A97V	rs17173703	0.533,P	C/T
129	GOLGA2	p.R686W	rs72756867	0.533,P	G/A
130	ALPK2	p.G810S	rs3809970	0.534,P	T/T
131	LILRA6	p.G149R	rs1052966	0.535,P	T/T
132	SDCCAG8	p.E77D	rs2275155	0.536,P	A/T
133	FAM86C1	p.A7S	rs12283300	0.537,P	T/T
134	TUBB8	p.H105R	rs9329307	0.537,P	T/C
135	MUC4	p.V1353A	rs12491056	0.539,P	G/G
136	PEMT	p.S222N	rs7946	0.539,P	T/T
137	EFCAB8	p.R118C	rs13037496	0.540,P	C/T
138	SPRR1A	p.V61I	rs1611764	0.542,P	G/A
139	GTPBP10	p.C88W	rs42663	0.542,P	T/G
140	TEKT4	p.T83M	rs4854235	0.542,P	C/T
141	ZNF768	p.E181D	rs10871453	0.543,P	C/G
142	UGT2A3	p.A497T	rs13128286	0.544,P	T/T
143	ABCB5	p.T131I	rs17143212	0.544,P	C/T
144	DCAF12	p.R131Q	rs11557154	0.545,P	C/T
145	LAMA5	p.R3079W	rs944895	0.545,P	G/A
146	ZNF653	p.E352K	rs74552618	0.546,P	C/T
147	CROCC	p.Q302E	rs143501240	0.547,P	C/G
148	VWDE	p.E869K	rs17165910	0.549,P	C/T
149	HIVEP1	p.T187M	rs2228209	0.549,P	C/T
150	MKI67	p.R2649H	rs12777740	0.550,P	C/T
151	TMEM244	p.F111V	rs7776426	0.550,P	A/C
152	GPR35	p.T284M	rs12468485	0.552,P	C/T
153	ZBTB42	p.E232K	rs4983387	0.552,P	G/A
154	EFHB	p.P139S	rs13078867	0.554,P	G/A
155	MUC6	p.T1822A	rs76686156	0.554,P	T/C
156	FMO2	p.E314G	rs2020863	0.555,P	A/G
157	FAM205A	p.T189M	rs1854574	0.556,P	G/A
158	ECM1	p.T130M	rs3737240	0.556,P	C/T
159	ITIH2	p.L569V	rs7084817	0.556,P	C/G
160	KANK3	p.E610K	rs7249069	0.556,P	C/T
161	ZNF808	p.D262N	rs329965	0.557,P	A/A
162	PIAS2	p.I598V	rs117151539	0.558,P	T/C
163	NCKAP5	p.N1093Y	rs16841277	0.558,P	T/A
164	TAS2R31	p.L48V	rs760444623	0.558,P	G/C
165	MKI67	p.T1247I	rs4750685	0.559,P	G/A
166	ZNF502	p.Q174R	rs56084453	0.559,P	G/G
167	OR11G2	p.I99N	rs4981822	0.560,P	A/A
168	SNTG2	p.S200L	rs6751090	0.560,P	C/T
169	FUT10	p.L368V	rs17855838	0.562,P	G/C

170	LILRB1	p.Y99N	rs570016342	**0.562,P**	T/A
171	DDX60L	p.V754A	rs61740705	**0.562,P**	A/G
172	TRIM49C	p.T394N	rs75119043	**0.562,P**	C/A
173	OBP2A	p.P133S	rs3178137	**0.563,P**	C/T
174	FHDC1	p.R639C	rs3811833	**0.563,P**	T/T
175	C1orf167	p.A850T	rs55967531	**0.563,P**	G/A
176	C9orf84	p.E1174G	rs7869279	**0.563,P**	T/C
177	CDHR2	p.L1164M	rs17078347	**0.564,P**	C/A
178	TAX1BP1	p.L307I	rs11540483	**0.566,P**	T/A
179	TMPRSS15	p.E134Q	rs2824790	**0.567,P**	C/G
180	NCAPG2	p.A132S	N/A	**0.569,P**	C/A
181	TNFAIP2	p.Q282E	rs1132339	**0.571,P**	C/G
182	HLA-DRB1	p.Y59H	rs11554462	**0.571,P**	A/G
183	SLC7A9	p.L223M	rs1007160	**0.574,P**	T/T
184	XRCC1	p.R194W	rs1799782	**0.574,P**	G/A
185	KRTAP10-10	p.V158M	rs4818950	**0.574,P**	G/A
186	WBSCR28	p.I14N	rs11770052	**0.576,P**	A/A
187	HLA-C	p.T187P	rs1050685	**0.577,P**	T/G
188	PIEZO1	p.I2265V	rs1803382	**0.578,P**	T/C
189	OR5B3	p.N170S	rs12280114	**0.580,P**	T/C
190	PCDHB16	p.T482I	rs17844646	**0.580,P**	C/T
191	MRPL9	p.E210A	rs8480	**0.581,P**	T/G
192	DSPP	p.D1074G	rs202210195	**0.583,P**	A/G
193	OSBPL1A	p.S810P	rs35693789	**0.583,P**	A/G
194	ADO	p.G25W	rs2236295	**0.584,P**	G/T
195	SIK3	p.P917R	rs12225230	**0.587,P**	G/C
196	MKI67	p.D2760G	rs10082391	**0.588,P**	T/C
197	MUC3A	p.T2827I	rs145584597	**0.588,P**	C/T
198	MUC4	p.H3245Q	rs1018195432	**0.589,P**	G/C
199	CASP12	p.T21I	rs138698464	**0.589,P**	G/A
200	SHBG	p.P69L	rs6258	**0.591,P**	C/T
201	SCLY	p.A183T	rs3210400	**0.593,P**	G/A
202	RNF32	p.R307C	rs2302146	**0.594,P**	C/T
203	CTU1	p.A107V	rs17855403	**0.595,P**	G/A
204	OR6C65	p.T222A	rs7971073	**0.595,P**	G/G
205	FAM171A1	p.P465S	rs3814165	**0.596,P**	A/A
206	CELA3B	p.R79W	rs7528405	**0.598,P**	T/T
207	GALP	p.I72M	rs3745833	**0.599,P**	C/G
208	EFHC1	p.R140W	rs3804506	**0.599,P**	C/T
209	SFI1	p.W330R	rs16989291	**0.600,P**	T/C
210	REG3A	p.H50P	rs201139260	**0.600,P**	T/G
211	PKD1L3	p.H571Q	rs1559401	**0.601,P**	T/T
212	OR52E2	p.N5S	rs16909440	**0.601,P**	T/C
213	10-Mar	p.F318S	rs9891498	**0.601,P**	A/G
214	INCENP	p.R697Q	rs116873148	**0.602,P**	G/A

215	MFSD9	p.I288T	rs33993717	**0.602,P**	A/G
216	KIR3DL1,KIR3DS1	p.W304L	rs35974949	**0.606,P**	G/T
217	ERBB2	p.P1155A	rs1058808	**0.607,P**	G/G
218	PZP	p.N857S	rs3213831	**0.607,P**	C/C
219	HNRNPA1L2	p.Y289C	rs78872760	**0.611,P**	A/G
220	TRIM51	p.N20S	rs2063276	**0.612,P**	A/G
221	SDAD1	p.S575C	rs2242471	**0.612,P**	G/C
222	GBP6	p.A331S	rs4658359	**0.612,P**	T/T
223	LRRC56	p.D523H	rs10902171	**0.615,P**	G/C
224	PTAFR	p.N114S	rs138629813	**0.615,P**	T/C
225	ZNF568	p.R461H	rs16971886	**0.615,P**	G/A
226	TOR1A	p.D216H	rs1801968	**0.615,P**	C/G
227	MAFA	p.G347C	rs62521874	**0.615,P**	A/A
228	SCARF1	p.E639D	rs3744644	**0.616,P**	G/G
229	OR52E6	p.S95P	rs4592451	**0.616,P**	A/G
230	KCNJ18	p.L211F	rs1435776313	**0.618,P**	C/T
231	TUBA3E	p.R221S	rs13000249	**0.619,P**	G/T
232	CDK5RAP2	p.E289Q	rs4836822	**0.619,P**	C/G
233	ACAT2	p.K211R	rs25683	**0.621,P**	G/G
234	KRTAP15-1	p.L43M	rs2832873	**0.622,P**	C/A
235	FAM83H	p.H437Y	rs28573699	**0.622,P**	G/A
236	OR14I1	p.D50N	rs4509608	**0.624,P**	T/T
237	OR10A4	p.R262Q	rs10839635	**0.627,P**	G/A
238	MRPL28	p.D160E	rs11557302	**0.628,P**	G/C
239	MPHOSPH6	p.I58V	rs2303267	**0.628,P**	T/C
240	OTOL1	p.E470A	rs3921595	**0.628,P**	A/C
241	S1PR2	p.V286A	rs117064827	**0.630,P**	A/G
242	TPR	p.S960N	rs3753565	**0.630,P**	C/T
243	EHBP1L1	p.V538G	rs6591182	**0.630,P**	T/G
244	COQ7	p.T103M	rs11074359	**0.631,P**	C/T
245	TMPRSS11E	p.Y303C	rs976002	**0.631,P**	A/G
246	UGT2B4	p.D322E	rs13119049	**0.633,P**	A/T
247	ERICH6	p.L426V	rs73003074	**0.633,P**	A/C
248	EYS	p.S2556C	rs66462731	**0.634,P**	T/A
249	TEX33	p.Y184C	rs9610624	**0.634,P**	T/C
250	FCER2	p.R62W	rs2228137	**0.636,P**	G/A
251	TTN	p.T26621M	rs3731746	**0.636,P**	G/A
252	ZNF669	p.E76D	rs4925692	**0.636,P**	C/A
253	RYR3	p.N2016S	rs942844610	**0.636,P**	A/G
254	HLA-DQB1	p.G77E	rs1049083	**0.637,P**	T/T
255	MROH5	p.A375V	rs12547980	**0.637,P**	A/A
256	IL1A	p.A114S	rs17561	**0.637,P**	C/A
257	STARD3	p.R117Q	rs1877031	**0.637,P**	A/A
258	NOC3L	p.E472A	rs3758526	**0.637,P**	T/G
259	GSDMA	p.T314N	rs56030650	**0.637,P**	C/A

260	RGS9BP	p.A96S	rs259290	**0.638,P**	G/T
261	PKD1L2	p.P301A	rs11150370	**0.639,P**	G/C
262	NOP9	p.S308N	rs4280164	**0.639,P**	G/A
263	AEN	p.S88C	rs8026929	**0.639,P**	C/G
264	MVB12A	p.D157N	rs35944915	**0.640,P**	G/A
265	RGPD3	p.E1287K	rs3898279	**0.640,P**	C/T
266	ZNF254	p.D52G	rs17854260	**0.641,P**	A/G
267	FBN3	p.P1958H	rs7245429	**0.641,P**	G/T
268	MT1A	p.T27N	rs11640851	**0.642,P**	A/A
269	MUC20	p.D322H	rs2688542	**0.642,P**	G/C
270	TSEN54	p.K347N	rs9911502	**0.642,P**	G/C
271	MKI67	p.R2786Q	rs10764749	**0.644,P**	C/T
272	KNG1	p.I197M	rs2304456	**0.644,P**	T/G
273	MUC6	p.P1794T	rs35549382	**0.645,P**	G/T
274	SLC52A1	p.V296M	rs2304445	**0.646,P**	C/T
275	PTCH1	p.P1164L	rs357564	**0.646,P**	G/A
276	ATAD5	p.E135G	rs11080134	**0.647,P**	A/G
277	DNAJC17	p.V204L	rs117485355	**0.647,P**	C/G
278	AHRR	p.P189A	rs2292596	**0.647,P**	C/G
279	TEX35	p.L171R	rs3813636	**0.648,P**	G/G
280	CCDC125	p.V13M	rs10471774	**0.649,P**	C/T
281	ECE2	p.R170W	rs11546878	**0.649,P**	T/T
282	GOLGA5	p.A67G	rs17128572	**0.650,P**	C/G
283	ABCG5	p.R50C	rs6756629	**0.650,P**	G/A
284	LILRB2	p.L243F	rs1473278082	**0.651,P**	G/A
285	MOCOS	p.S22R	rs113873219	**0.652,P**	C/A
286	C10orf11	p.S181F	rs35349706	**0.652,P**	T/T
287	KBTBD13	p.A81V	rs2919358	**0.653,P**	C/T
288	CDON	p.A686V	rs12274923	**0.654,P**	G/A
289	CRTC1	p.T344A	rs3746266	**0.654,P**	A/G
290	OR52N1	p.T79N	rs12365487	**0.655,P**	T/T
291	GOLGA6L2	p.R235P	rs12594944	**0.655,P**	G/G
292	PLB1	p.A1318V	rs2199619	**0.655,P**	C/T
293	TSEN54	p.I137L	rs11559205	**0.656,P**	A/C
294	FLG2	p.C298S	rs2282302	**0.656,P**	C/G
295	KIF20B	p.K1609E	rs34354493	**0.656,P**	A/G
296	OR51I2	p.R151P	rs16931292	**0.657,P**	G/C
297	OR5L2	p.R141P	rs75822385	**0.657,P**	G/C
298	TAS2R43	p.L235F	rs3759244	**0.658,P**	A/A
299	LAMA4	p.R538C	rs138153075	**0.659,P**	G/A
300	HLA-DPB1	p.L207M	rs14362	**0.659,P**	C/A
301	WDYHV1	p.R66C	rs3824250	**0.659,P**	C/T
302	NCAPD2	p.V797M	rs10849482	**0.660,P**	G/A
303	CRYBG3	p.S372F	rs17301717	**0.660,P**	T/T
304	VCAN	p.G428D	rs2287926	**0.660,P**	A/A

305	DNAH14	p.N1099Y	rs3128655	**0.660,P**	A/T
306	COASY	p.S84Y	rs615942	**0.660,P**	C/A
307	AHNAK2	p.P1562L	rs61996045	**0.660,P**	A/A
308	FAM220A	p.R127Q	rs3750040	**0.662,P**	C/T
309	OR52R1	p.I129T	rs7941731	**0.662,P**	A/G
310	OTOP2	p.G465W	rs6501741	**0.663,P**	G/T
311	UBASH3B	p.A68T	rs12790613	**0.665,P**	G/A
312	KRTAP12-2	p.S29C	rs7275281	**0.665,P**	G/C
313	DNAJB11	p.I264V	rs8147	**0.665,P**	A/G
314	HLA-DQB2	p.S228G	rs9276572	**0.665,P**	C/C
315	C7	p.S389T	rs1063499	**0.666,P**	C/C
316	FXYD4	p.D28H	rs150156235	**0.669,P**	G/C
317	ZNF493	p.L420V	rs10414834	**0.669,P**	C/G
318	TLR10	p.N241H	rs11096957	**0.669,P**	T/G
319	PKD1L2	p.A2055T	rs16954717	**0.669,P**	C/T
320	RNF39	p.D268N	rs1057539	**0.670,P**	C/T
321	OR9G1,OR9G9	p.R169C	rs11228733	**0.670,P**	C/T
322	DNAH11	p.S654C	rs62441683	**0.670,P**	C/G
323	MAP2K3	p.R65L	rs56067280	**0.671,P**	G/T
324	GBP1	p.A409G	rs1048443	**0.672,P**	G/C
325	AHNAK2	p.P5397A	rs3742935	**0.672,P**	C/C
326	KRT6C	p.G111D	rs394598	**0.672,P**	T/T
327	OR1L6	p.I215T	rs10985760	**0.673,P**	T/C
328	C14orf159	p.D507N	rs2295524	**0.673,P**	G/A
329	GPR101	p.V124L	rs1190736	**0.674,P**	A/A
330	LRMP	p.C197S	rs1908946	**0.674,P**	G/C
331	GLMP	p.P203S	rs10908496	**0.675,P**	G/A
332	NIPSNAP3A	p.R100Q	rs2274870	**0.675,P**	G/A
333	CHMP4A	p.G196R	rs2295322	**0.675,P**	C/T
334	BPIFB4	p.N320T	rs2889732	**0.675,P**	A/C
335	HLA-C	p.T187M	rs1050686	**0.676,P**	G/A
336	TIGD2	p.H475R	rs2280099	**0.677,P**	A/G
337	FCGBP	p.P1961S	rs1464897604	**0.679,P**	G/A
338	COL11A2	p.P894L	rs2855430	**0.680,P**	G/A
339	NDUFAF1	p.A314G	rs12900702	**0.683,P**	G/C
340	PTPRB	p.G2152A	rs17226367	**0.683,P**	C/G
341	PROM2	p.G513S	rs72819488	**0.684,P**	G/A
342	IGFN1	p.S3069R	rs61743921	**0.686,P**	C/A
343	HSD17B4	p.R82H	rs25640	**0.687,P**	G/A
344	CTTNBP2	p.L1213V	rs62617115	**0.687,P**	A/C
345	ZNF717	p.R350C	rs1962893	**0.688,P**	G/A
346	WDR18	p.I62V	rs61732720	**0.690,P**	A/G
347	ALS2CL	p.E45Q	rs7642448	**0.690,P**	C/G
348	URAD	p.R114S	rs9579139	**0.690,P**	G/T
349	USP31	p.R931L	rs10083789	**0.691,P**	C/A

350	CWH43	p.H689N	rs1051447	**0.691,P**	A/A
351	R3HCC1	p.L321R	rs13530	**0.691,P**	G/G
352	TM6SF1	p.L48R	rs79470022	**0.691,P**	T/G
353	RANBP3L	p.V451D	rs145254521	**0.692,P**	A/T
354	PLAU	p.L141P	rs2227564	**0.694,P**	C/C
355	IL27RA	p.L188P	rs35026308	**0.694,P**	T/C
356	SH3TC2	p.M1184V	rs142451273	**0.695,P**	T/C
357	POM121	p.Ala7Glu	rs143666868	**0.695,P**	C/A
358	LRRC17	p.K119E	rs3800939	**0.696,P**	A/G
359	KLK11	p.R166C	rs1048328	**0.697,P**	G/A
360	DOK3	p.P294R	rs61749657	**0.697,P**	G/C
361	VPS13D	p.S3777C	rs149521489	**0.698,P**	C/G
362	NECAB1	p.A271S	rs115555424	**0.700,P**	G/T
363	FGFR4	p.G388R	rs351855	**0.700,P**	G/A
364	TNC	p.R322Q	rs145315080	**0.701,P**	C/T
365	OR2M7	p.V78A	rs7555310	**0.701,P**	A/G
366	MKI67	p.N104S	rs2071498	**0.702,P**	C/C
367	TRIM49	p.G373R	rs12417980	**0.703,P**	T/T
368	PLEKHG5	p.E794K	rs184242303	**0.703,P**	C/T
369	TNN	p.P930L	rs2285215	**0.703,P**	T/T
370	SAXO2	p.P47L	rs16973457	**0.704,P**	C/T
371	ACACB	p.E1815K	rs61752535	**0.704,P**	A/A
372	VCAN	p.D2937Y	rs160277	**0.706,P**	G/T
373	ZNF254	p.K496N	rs12611425	**0.707,P**	G/C
374	AHNAK2	p.P2387S	rs72702027	**0.708,P**	A/A
375	MEIG1	p.K9T	rs4750568	**0.709,P**	C/C
376	TMED5	p.T175I	rs1060622	**0.710,P**	A/A
377	PLCXD1	p.G305V	rs112920828	**0.711,P**	G/T
378	CA6	p.S90G	rs2274333	**0.713,P**	A/G
379	MYH6	p.A1130T	rs28730771	**0.713,P**	C/T
380	CLEC4A	p.H36L	rs2024301	**0.714,P**	A/T
381	POLL	p.R438W	rs3730477	**0.714,P**	A/A
382	C14orf37	p.T96I	rs3829765	**0.714,P**	G/A
383	GRIN2C	p.L65F	rs78349823	**0.714,P**	G/A
384	SNX31	p.D73H	rs2187016	**0.715,P**	C/G
385	CA6	p.T55M	rs2274327	**0.715,P**	T/T
386	ABCG8	p.D19H	rs11887534	**0.717,P**	G/C
387	C1orf27	p.S251C	rs12084264	**0.717,P**	C/G
388	TAS2R19	p.F290S	rs72475480	**0.718,P**	A/G
389	MAPKBP1	p.V392I	rs75869993	**0.718,P**	G/A
390	OR5T2	p.A195T	rs77295387	**0.718,P**	C/T
391	MYOM1	p.S181P	rs1962519	**0.719,P**	G/G
392	MYO10	p.R324W	rs11750538	**0.720,P**	A/A
393	TJP3	p.R28H	rs2067019	**0.720,P**	G/A
394	DCLRE1A	p.D317H	rs3750898	**0.720,P**	C/G

395	TGM5	p.R81C	rs773076654	**0.720,P**	G/A
396	NLRP14	p.E808K	rs10839708	**0.721,P**	A/A
397	TTN	p.V3261M	rs2291311	**0.721,P**	T/T
398	SSX5	p.E19Q	rs4824675	**0.721,P**	G/G
399	DMRT2	p.E458Q	rs17641078	**0.723,P**	C/C
400	OSBPL10	p.N254D	rs2290532	**0.724,P**	T/C
401	NPAP1	p.V212A	rs3784246	**0.725,P**	T/C
402	HK2	p.R373W	rs199992983	**0.728,P**	C/T
403	CDH4	p.G523S	rs142900721	**0.729,P**	G/A
404	CSMD3	p.N3581H	rs1592624	**0.730,P**	G/G
405	GAK	p.D787Y	rs34585705	**0.730,P**	C/A
406	SIGLEC6	p.P246S	rs2305772	**0.732,P**	A/A
407	KCNJ12	p.S371R	rs1612176	**0.734,P**	C/G
408	DNAH9	p.N2195S	rs3744581	**0.734,P**	A/G
409	SERPINA7	p.L303F	rs1804495	**0.735,P**	A/A
410	OR6M1	p.T276K	rs4936845	**0.735,P**	T/T
411	ANGPTL8	p.R59W	rs2278426	**0.736,P**	C/T
412	KRTAP6-1	p.R43C	rs74914596	**0.736,P**	G/A
413	RTL1	p.E848Q	rs11623267	**0.737,P**	C/G
414	TCHH	p.L790M	rs11803731	**0.737,P**	T/T
415	ACOT4	p.R57C	rs3742819	**0.738,P**	C/T
416	TRPA1	p.E179K	rs920829	**0.739,P**	C/T
417	ERICH6B	p.V653F	rs1536207	**0.740,P**	C/A
418	DNAH1	p.V3406I	rs201752275	**0.740,P**	G/A
419	RP1L1	p.H222P	rs4388421	**0.740,P**	G/G
420	ARHGEF28	p.R585K	rs2973566	**0.744,P**	G/A
421	DLGAP4	p.R747Q	rs41274714	**0.744,P**	G/A
422	CP	p.T841R	rs56033670	**0.744,P**	G/C
423	OR51I1	p.R302C	rs61736831	**0.745,P**	G/A
424	C9orf173	p.L218F	rs61759822	**0.745,P**	T/T
425	OR7A10	p.L44I	rs12985894	**0.746,P**	G/T
426	IMMT	p.P124S	rs1050301	**0.747,P**	G/A
427	ZNF234	p.V420I	rs201556875	**0.747,P**	G/A
428	KIAA0753	p.P566L	rs2304977	**0.747,P**	A/A
429	YY1AP1	p.D231N	rs41264945	**0.747,P**	C/T
430	C2orf74	p.Y37D	rs1729674	**0.748,P**	T/G
431	MAP7	p.R580W	rs2076190	**0.751,P**	A/A
432	ZNF681	p.K336R	rs7245561	**0.751,P**	T/C
433	HTR3D	p.R225H	rs1000952	**0.753,P**	A/A
434	ALNT4,POC1B-GALN	p.V334I	rs2230283	**0.753,P**	C/T
435	TET2	p.L1721W	rs34402524	**0.754,P**	T/G
436	ZNF19	p.Q218H	rs8050871	**0.754,P**	G/G
437	UQCRH	p.E22G	rs41292543	**0.755,P**	A/G
438	USP6	p.R912Q	rs9899177	**0.755,P**	G/A
439	RASIP1	p.R601C	rs2287922	**0.756,P**	A/A

440	CERS2	p.E115A	rs267738	**0.756,P**	T/G
441	KIR3DL1,KIR3DS1	p.I75L	rs1049150	**0.757,P**	A/T
442	SH3RF3	p.A15T	rs34609468	**0.757,P**	G/A
443	VPS41	p.E432K	rs62444122	**0.757,P**	C/T
444	TAS2R38	p.I296V	rs10246939	**0.759,P**	T/C
445	SLC39A8	p.L449F	rs112519623	**0.759,P**	G/A
446	POU5F1B	p.G176E	rs6998061	**0.759,P**	G/A
447	COL6A6	p.I916T	rs61730505	**0.760,P**	T/C
448	SFTPC	p.T138N	rs4715	**0.761,P**	C/A
449	LILRA1	p.V387L	rs116973751	**0.764,P**	G/T
450	OR13C5	p.S18F	rs1851722	**0.764,P**	G/A
451	AQP12B	p.R23Q	rs4081909	**0.764,P**	C/T
452	CATSPERD	p.G639R	rs61180947	**0.764,P**	G/A
453	CPN1	p.G178D	rs61751507	**0.764,P**	C/T
454	OR2Y1	p.V200L	rs10464105	**0.765,P**	G/G
455	OR4A47	p.S236P	rs76991989	**0.765,P**	T/C
456	PNPLA2	p.L481P	rs1138693	**0.766,P**	C/C
457	DDX58	p.D580E	rs17217280	**0.766,P**	A/T
458	SLCO5A1	p.G594V	rs34698405	**0.767,P**	C/A
459	IGSF10	p.Y150D	rs7619322	**0.768,P**	C/C
460	HLA-A	p.P217A	rs1059563	**0.769,P**	C/G
461	PPP2R3B	p.A519V	rs1133520	**0.769,P**	G/A
462	TPCN2	p.V219I	rs72928978	**0.769,P**	G/A
463	FHL2	p.T171M	rs727504674	**0.770,P**	G/A
464	KRT76	p.A359T	rs6580904	**0.771,P**	C/T
465	EPG5	p.S1083L	rs78339727	**0.771,P**	G/A
466	NQO1	p.R139W	rs1131341	**0.773,P**	G/A
467	OPRM1	p.N133D	rs1799971	**0.775,P**	A/G
468	C2orf16	p.I774V	rs1919128	**0.775,P**	A/G
469	OTOP3	p.M1T	rs368367098	**0.775,P**	T/C
470	PNMAL2	p.V401I	rs3745790	**0.775,P**	C/T
471	IL37	p.G31V	rs3811046	**0.775,P**	G/T
472	MRGPRE	p.G160S	rs4391795	**0.776,P**	C/T
473	KIR3DL1	p.C364Y	rs45542639	**0.776,P**	G/A
474	PSMB4	p.I234T	rs4603	**0.776,P**	T/C
475	SDCBP2	p.R223C	rs1048621	**0.777,P**	A/A
476	OR7C1	p.V247L	rs73004304	**0.777,P**	C/G
477	GALNT9	p.A400T	rs11246991	**0.778,P**	C/T
478	VCAN	p.K349E	rs61749613	**0.778,P**	A/G
479	ALKBH3	p.D228E	rs1130290	**0.779,P**	C/G
480	ACAN	p.S939T	rs938609	**0.779,P**	T/A
481	MAVS	p.Q198K	rs7262903	**0.780,P**	C/A
482	CORO2B	p.L189Q	rs138122643	**0.781,P**	T/A
483	TRPM8	p.Y251C	rs17868387	**0.781,P**	A/G
484	SMYD4	p.P797H	rs58337165	**0.781,P**	G/T

485	NRAP	p.N519I	rs2270182	**0.783,P**	T/A
486	OXCT2	p.D494N	rs150795467	**0.784,P**	C/T
487	ALG9	p.V289I	rs10502151	**0.785,P**	C/T
488	FAM35A	p.S550C	rs11202365	**0.785,P**	T/T
489	KIF9	p.R638W	rs2276853	**0.785,P**	A/A
490	MUC20	p.A296T	rs572650436	**0.785,P**	G/A
491	POM121L12	p.A3S	rs72598684	**0.785,P**	T/T
492	ZFR2	p.R521C	rs61742027	**0.786,P**	G/A
493	TRAPPC12	p.S301G	rs11686212	**0.787,P**	A/G
494	DDX51	p.Q295R	rs1133690	**0.788,P**	C/C
495	HLA-DRB5	p.D40H	rs1136752	**0.788,P**	C/G
496	SVIL	p.L1064R	rs199726033	**0.789,P**	A/C
497	MKI67	p.T2868S	rs2071496	**0.789,P**	G/C
498	CCDC93	p.R179C	rs33975708	**0.790,P**	G/A
499	CAMK2N2	p.K47T	rs1332780006	**0.792,P**	T/G
500	GEMIN5	p.R1016C	rs61749643	**0.792,P**	G/A
501	SPATA31A6	p.P1102L	rs10907643	**0.794,P**	T/T
502	ATF7IP	p.K538R	rs3213764	**0.794,P**	G/G
503	ITGA11	p.P972L	rs4777035	**0.794,P**	A/A
504	CABLES2	p.V381L	rs61742254	**0.794,P**	C/G
505	KCNMB1	p.A27V	N/A	**0.797,P**	G/A
506	EVC	p.D95G	rs41269547	**0.797,P**	A/G
507	GRIN3A	p.G487R	rs10989589	**0.799,P**	C/T
508	MKI67	p.E497D	rs11016076	**0.799,P**	C/G
509	HSPB9	p.Q2P	rs1122326	**0.799,P**	A/C
510	NINJ1	p.A110D	rs2275848	**0.799,P**	G/T
511	DISC1	p.S736C	rs821616	**0.799,P**	A/T
512	MUC16	p.T2506A	rs1609458	**0.801,P**	T/C
513	ALPK1	p.G565D	rs2074388	**0.801,P**	G/A
514	KLRB1	p.I168T	rs1135816	**0.802,P**	G/G
515	GFRA2	p.L462Q	rs1128397	**0.804,P**	A/T
516	WBSCR27	p.S171W	rs13232463	**0.804,P**	C/C
517	ESYT2	p.S584P	rs2305475	**0.804,P**	A/G
518	TICRR	p.R1885C	rs3743372	**0.804,P**	C/T
519	SEC16B	p.Q845H	rs7522194	**0.804,P**	C/A
520	C2CD2L	p.S466T	rs1946807141	**0.805,P**	T/A
521	PCDHGB3	p.L715M	rs138338803	**0.805,P**	C/A
522	KIF24	p.W218L	rs17350674	**0.806,P**	C/A
523	SARDH	p.R614H	rs2073817	**0.806,P**	C/T
524	ADGRF2	p.L256M	rs17541107	**0.807,P**	T/A
525	P2RX7	p.Y155H	rs208294	**0.808,P**	T/C
526	CR1L	p.L491P	rs2796257	**0.808,P**	T/C
527	SMYD4	p.R562W	rs11549830	**0.809,P**	G/A
528	MMS22L	p.T564M	rs9481410	**0.809,P**	G/A
529	ANXA2R	p.Q119R	rs1054428	**0.810,P**	C/C

530	MAGEC3	p.A328T	rs176026	**0.811,P**	A/A
531	KRT13	p.A187V	rs9891361	**0.811,P**	A/A
532	VTA1	p.C38S	rs2232300	**0.812,P**	T/A
533	MUS81	p.R37H	rs13817	**0.813,P**	G/A
534	SACS	p.N232K	rs2031640	**0.813,P**	A/T
535	PLA2G7	p.V379A	rs1051931	**0.814,P**	G/G
536	TUBGCP6	p.S1364C	rs5771107	**0.815,P**	T/A
537	SLC6A15	p.E684D	rs145111717	**0.816,P**	C/A
538	OR5V1	p.L23W	rs6930033	**0.817,P**	A/C
539	OR13C5	p.L69M	rs7042502	**0.817,P**	A/T
540	RET	p.R982C	rs17158558	**0.818,P**	C/T
541	TNXB	p.N4055I	rs17421133	**0.819,P**	T/A
542	KRT40	p.E286D	rs721958	**0.819,P**	G/G
543	MUC16	p.S1953P	rs1108380	**0.820,P**	A/G
544	HJURP	p.S549C	rs3821238	**0.821,P**	G/C
545	BCLAF1	p.S209C	rs6940018	**0.823,P**	G/C
546	NPW	p.E100Q	rs11248906	**0.824,P**	C/C
547	ALMS1	p.S2101L	rs28730854	**0.824,P**	C/T
548	IZUMO4	p.Y137F	rs45506200	**0.824,P**	A/T
549	ZAN	p.A2511V	rs76325149	**0.824,P**	C/T
550	KLHL38	p.C504Y	rs11779866	**0.825,P**	C/T
551	CFB	p.R32W	rs12614	**0.825,P**	C/T
552	ITGAE	p.R950W	rs1716	**0.825,P**	G/A
553	PCDHB2	p.F516L	rs143150465	**0.826,P**	C/A
554	FOXA1	p.E269V	rs757337935	**0.827,P**	T/A
555	PRTN3	p.R249H	rs150802678	**0.828,P**	G/A
556	NKAIN4	p.A131D	rs2236194	**0.828,P**	G/T
557	EXOC3L4	p.R77W	rs2297067	**0.828,P**	C/T
558	PLXNA2	p.E369G	rs4844658	**0.828,P**	T/C
559	OR51B5	p.T78K	rs57273781	**0.828,P**	G/T
560	EYA4	p.G277S	rs9493627	**0.828,P**	G/A
561	TPSG1	p.R193H	rs143120059	**0.829,P**	T/T
562	MUC4	p.N3782K	rs2550240	**0.829,P**	C/C
563	NACAD	p.C1152F	rs3735493	**0.829,P**	C/A
564	SPATA31E1	p.D1202G	rs11789780	**0.830,P**	G/G
565	SERPINB11	p.A181T	rs1506418	**0.830,P**	G/A
566	PYCRL	p.V117M	rs2242089	**0.830,P**	C/T
567	MLIP	p.S855T	rs6934690	**0.831,P**	A/A
568	DNHD1	p.V2374M	rs11606889	**0.832,P**	G/A
569	SH2D1B	p.N122K	rs34001279	**0.832,P**	G/T
570	ODF4	p.R77C	rs73250854	**0.833,P**	C/T
571	CEP131	p.A1020T	rs117616373	**0.834,P**	C/T
572	SLC25A5	p.I79F	rs141428607	**0.834,P**	A/T
573	ENDOD1	p.G446V	rs3740861	**0.834,P**	G/T
574	GOLGA6L2	p.R317W	rs59122400	**0.835,P**	A/A

575	OR8A1	p.T133R	rs55861866	**0.836,P**	C/G
576	APOL5	p.T323M	rs2076672	**0.837,P**	C/T
577	OR2M2	p.R220G	rs4244171	**0.838,P**	C/G
578	KRT75	p.M438T	rs764819403	**0.838,P**	A/G
579	TCOF1	p.P1176R	rs1136103	**0.839,P**	C/G
580	EYS	p.E641V	rs17411795	**0.839,P**	T/A
581	HLA-A	p.E277Q	rs2231095	**0.840,P**	C/C
582	ERCC6L2	p.V592A	rs2274654	**0.840,P**	T/C
583	ACAN	p.S930I	rs938608	**0.840,P**	G/T
584	OR10J1	p.I103M	rs12048482	**0.841,P**	A/G
585	EML5	p.I269V	rs17188228	**0.841,P**	T/C
586	ACADL	p.K333Q	rs2286963	**0.841,P**	T/G
587	PRR29	p.T24S	rs62070903	**0.841,P**	C/G
588	GRWD1	p.A64V	rs1971402770	**0.842,P**	C/T
589	CDH23	p.G490A	rs1227049	**0.842,P**	G/C
590	MS4A15	p.S20G	rs12363342	**0.842,P**	A/G
591	NEK4	p.P225A	rs1029871	**0.843,P**	G/C
592	HLA-DQA1	p.R70W	rs1142326	**0.843,P**	T/T
593	OR10A6	p.V140G	rs7933807	**0.843,P**	A/C
594	TGOLN2	p.R259W	rs4247303	**0.844,P**	G/A
595	CARD6	p.S86L	rs10512747	**0.845,P**	C/T
596	TAS2R19	p.C264Y	rs76970958	**0.845,P**	C/T
597	ECHDC3	p.A69T	rs4750090	**0.846,P**	A/A
598	RPL22L1	p.V96F	rs13462	**0.847,P**	C/A
599	INMT	p.E219G	rs2302340	**0.847,P**	A/G
600	SPANXB1	p.D86H	rs1218473051	**0.848,P**	G/C
601	C21orf58	p.P194S	rs13047478	**0.848,P**	G/A
602	MS4A6E	p.V47F	rs2304933	**0.849,P**	G/T
603	CRTC3	p.S72N	rs8033595	**0.850,P**	G/A
604	FMN2	p.P962L	rs111896385	**0.851,P**	T/T
605	MEFV	p.E148Q	rs3743930	**0.851,P**	C/G
606	NT5C3B	p.S213C	rs1046404	**0.852,P**	C/C
607	LYSMD4	p.A181G	rs2061007	**0.852,P**	G/C
608	PRR22	p.P248L	rs34572934	**0.852,P**	G/A
609	ZNF705A	p.K142Q	rs10743253	**0.854,P**	A/C
610	KRT82	p.T458M	rs2658658	**0.854,P**	G/A
611	OBSCN	p.A3729T	rs437129	**0.855,P**	G/A
612	ANKRD62	p.E406K	rs4519391	**0.855,P**	A/A
613	NEB	p.K1027N	rs6735208	**0.855,P**	A/A
614	ZNF880	p.V12M	rs14048	**0.856,P**	G/A
615	BANK1	p.A383T	rs3733197	**0.856,P**	G/A
616	C3orf18	p.A162V	rs1034405	**0.857,P**	A/A
617	PALD1	p.R721C	rs3740447	**0.857,P**	C/T
618	BRWD1	p.S1511P	rs2183573	**0.858,P**	G/G
619	CTBP2	p.L392P	rs3781412	**0.858,P**	A/G

620	CCDC183	p.L113R	rs4546744	**0.858,P**	T/G
621	MLH3	p.R1152C	rs569011240	**0.858,P**	G/A
622	DOCK8	p.R1238H	rs767874435	**0.858,P**	G/A
623	CCDC96	p.Q231H	rs374259671	**0.859,P**	C/G
624	SHROOM1	p.T488N	rs769565321	**0.859,P**	G/T
625	SMTNL1	p.R345G	rs12223229	**0.859,P**	C/G
626	PLEKHG4B	p.R1502G	rs12519352	**0.859,P**	C/G
627	RREB1	p.G195R	rs1334576	**0.859,P**	G/A
628	CCDC34	p.E264A	rs17244028	**0.860,P**	T/G
629	APOL1	p.E166K	rs2239785	**0.860,P**	A/A
630	OR10P1	p.V200M	rs7970885	**0.860,P**	G/A
631	CLEC18A	p.T151M	rs75776403	**0.861,P**	C/T
632	OBSCN	p.V3149M	rs1188697	**0.862,P**	G/A
633	HLA-DRB5	p.G30V	rs146966122	**0.862,P**	C/A
634	USP35	p.V236M	rs2510044	**0.862,P**	G/A
635	PLEKHG4B	p.R1432Q	rs4956987	**0.862,P**	G/A
636	TTC29	p.A302T	rs10013280	**0.863,P**	C/T
637	ZNF208	p.E282K	rs2007506	**0.863,P**	C/T
638	PKD1L2	p.L2119I	rs8050204	**0.863,P**	G/T
639	MYH7B	p.L1663F	rs1319423309	**0.864,P**	C/T
640	OR5B3	p.A181T	rs11229411	**0.864,P**	C/T
641	RNASEL	p.R462Q	rs486907	**0.864,P**	T/T
642	OR10H5	p.A224D	rs61745514	**0.864,P**	C/A
643	HLA-A	p.I166T	rs1059516	**0.865,P**	T/C
644	HELT	p.L62V	rs1078461	**0.866,P**	C/G
645	OR51M1	p.L257R	rs2736531	**0.866,P**	T/G
646	AMPH	p.K496T	rs35024632	**0.866,P**	T/G
647	ERV3-1	p.N569S	rs4717229	**0.866,P**	C/C
648	S100P	p.V14I	rs187031070	**0.867,P**	G/A
649	CSNK2A3	p.I133T	rs2071460	**0.867,P**	G/G
650	TRIML1	p.E132K	rs13131525	**0.868,P**	G/A
651	OSCAR	p.C105W	rs1488511419	**0.870,P**	G/C
652	CST9	p.L48F	rs2983640	**0.870,P**	G/A
653	BMP2	p.R190S	rs235768	**0.871,P**	T/T
654	SENP7	p.Q612H	rs2433031	**0.871,P**	T/A
655	ZNF750	p.P288L	rs35653278	**0.871,P**	G/A
656	DHDH	p.G282R	rs3765148	**0.871,P**	G/A
657	HLA-A	p.P208A	rs1136741	**0.872,P**	C/G
658	PLEKHH1	p.G677R	rs61534804	**0.872,P**	G/A
659	ARHGEF4	p.R650C	rs61758709	**0.872,P**	C/T
660	NLRP3	p.L331V	rs202077909	**0.873,P**	C/G
661	ATP10A	p.T532M	rs2066703	**0.873,P**	G/A
662	CTBP2	p.R298Q	rs3781411	**0.873,P**	C/T
663	OR12D2	p.V47F	rs9257834	**0.873,P**	G/T
664	COL6A3	p.E1386K	rs146092501	**0.874,P**	C/T

665	CCDC141	p.E382D	rs34883828	**0.875,P**	C/A
666	EPSTI1	p.N399K	rs1044856	**0.876,P**	A/T
667	SPATA24	p.E176E	rs10900862	**0.876,P**	T/T
668	TPSD1	p.A127V	rs143993373	**0.876,P**	C/T
669	OR2D2	p.S148P	rs1965209	**0.876,P**	A/G
670	KCNJ12	p.E378K	rs78547883	**0.876,P**	G/A
671	TTN	p.R24947C	rs744426	**0.877,P**	G/A
672	RP1L1	p.E2070V	rs11782670	**0.878,P**	A/A
673	C6orf118	p.I256M	rs510579	**0.878,P**	A/C
674	SPEG	p.P2687T	rs13026308	**0.879,P**	C/A
675	FLT3	p.T227M	rs1933437	**0.879,P**	G/A
676	SYNPO2L	p.G2S	rs60632610	**0.879,P**	C/T
677	MTCH2	p.P290A	rs1064608	**0.881,P**	G/C
678	DUPD1	p.D66N	rs11594934	**0.881,P**	C/T
679	ZSCAN5A	p.G337V	rs34187696	**0.881,P**	C/A
680	TMEM99	p.L95R	rs1044806	**0.883,P**	T/G
681	PKD1L3	p.T669S	rs35259348	**0.883,P**	G/C
682	ADGRV1	p.Y2232C	rs10037067	**0.885,P**	G/G
683	AP5B1	p.G170V	rs12362011	**0.885,P**	C/A
684	HLA-DRB5	p.Q39L	rs201948867	**0.885,P**	T/A
685	TPD52L3	p.F118L	rs3847262	**0.885,P**	C/C
686	LRP2	p.G4417D	rs41268685	**0.885,P**	C/T
687	PEAR1	p.S381F	rs77795865	**0.885,P**	C/T
688	OR51F1	p.A251V	rs17324609	**0.886,P**	G/A
689	COL6A6	p.A370T	rs9830253	**0.886,P**	A/A
690	HSPA1L	p.E602K	rs2075800	**0.887,P**	C/T
691	PNPLA7	p.W899S	rs61747535	**0.887,P**	C/G
692	PRKAG1	p.T89S	rs1126930	**0.888,P**	G/C
693	MTDH	p.A78S	rs17854373	**0.888,P**	G/T
694	UBAP2	p.R14Q	rs1785506	**0.889,P**	C/T
695	ERAP1	p.K528R	rs30187	**0.889,P**	T/C
696	CEP128	p.H732R	rs327463	**0.890,P**	T/C
697	ALPK2	p.K829N	rs3809973	**0.891,P**	G/G
698	C5orf60	p.W240R	rs62405726	**0.891,P**	A/G
699	OR11L1	p.G108S	rs10888257	**0.892,P**	T/T
700	AJAP1	p.G263R	rs242056	**0.892,P**	A/A
701	OLFML1	p.T53M	rs141351486	**0.893,P**	C/T
702	ARHGAP1	p.G183E	rs775108640	**0.893,P**	C/T
703	KHDC3L	p.A201G	rs561930	**0.894,P**	G/G
704	KRTAP6-3	p.Y51S	rs9305426	**0.894,P**	A/C
705	HLA-A	p.R169H	rs1059520	**0.895,P**	G/A
706	MUC16	p.R1015G	rs17000950	**0.895,P**	T/C
707	TCEB3B	p.A446T	rs3744863	**0.895,P**	C/T
708	KRTAP10-11	p.R269C	rs462007	**0.895,P**	T/T
709	ESPL1	p.S614R	rs1318648	**0.896,P**	C/A

710	PSPH	p.G90S	rs75395437	**0.896,P**	C/T
711	LIPF	p.T138A	rs814628	**0.896,P**	A/G
712	TTYH2	p.A409E	rs9892705	**0.896,P**	C/A
713	PCDHGB5	p.S594L	rs141997055	**0.897,P**	C/T
714	GHR	p.I544L	rs6180	**0.897,P**	C/C
715	ADGRF2	p.S49P	rs13212023	**0.898,P**	T/C
716	MRM1	p.C120S	rs78943308	**0.898,P**	G/C
717	OR10AD1	p.Y279H	rs11168459	**0.899,P**	A/G
718	KRR1	p.R134Q	rs11540407	**0.899,P**	T/T
719	OR9G9,OR9G1	p.G193C	rs12421330	**0.899,P**	G/T
720	TMEM41A	p.G236E	rs139252054	**0.901,P**	C/T
721	ZNF736	p.A209E	rs11976837	**0.902,P**	C/A
722	RNF43	p.R117H	rs2257205	**0.902,P**	C/T
723	MEGF6	p.G1137A	rs4648506	**0.902,P**	C/G
724	HLA-DRB1	p.R42W	rs1136758	**0.903,P**	A/A
725	GIPR	p.E354Q	rs1800437	**0.904,P**	C/C
726	TUBA3E	p.R156W	rs62165074	**0.905,P**	G/A
727	CELSR3	p.Q331R	rs1051589441	**0.906,P**	T/C
728	SGK223	p.G1226S	rs13269488	**0.906,P**	C/T
729	ALPK2	p.H1174P	rs3809977	**0.906,P**	G/G
730	SIM2	p.L483M	rs2073601	**0.908,P**	A/A
731	CDRT1	p.N644H	rs79385100	**0.908,P**	T/G
732	RNMTL1	p.V339D	rs80220493	**0.908,P**	T/A
733	AHNAK2	p.P3336L	rs10438247	**0.909,D**	A/A
734	OTOGL	p.N2237S	rs1551122	**0.910,D**	G/G
735	ADH7	p.G100A	rs1573496	**0.910,D**	C/G
736	KRT1	p.A454S	rs17678945	**0.910,D**	C/A
737	WSCD2	p.T266I	rs3764002	**0.910,D**	C/T
738	PCDH15	p.D440A	rs4935502	**0.910,D**	T/G
739	ANGEL1	p.F515C	rs2075773	**0.911,D**	A/C
740	DNAH12	p.D1572N	rs6773904	**0.911,D**	C/T
741	RBM46	p.I126M	rs79167802	**0.911,D**	T/G
742	SNAPC2	p.L118V	rs475002	**0.912,D**	C/G
743	MAN2B2	p.T892M	rs61729241	**0.912,D**	C/T
744	MUC3A	p.P2890L	rs144580177	**0.913,D**	C/T
745	PGPEP1L	p.P3L	rs2593051	**0.913,D**	A/A
746	MYO15A	p.Y2682F	rs712270	**0.913,D**	A/T
747	FAM186A	p.K187Q	rs12303082	**0.914,D**	T/G
748	ZNF208	p.P452T	rs12975751	**0.914,D**	G/T
749	GOLGA4	p.Q1050K	rs11718848	**0.915,D**	C/A
750	HLA-DRB1	p.T80R	rs1059582	**0.916,D**	G/C
751	FBLIM1	p.S191F	rs10927851	**0.916,D**	C/T
752	CASC5	p.K1285E	rs17747633	**0.916,D**	A/G
753	SLC35G5	p.G46D	rs6990563	**0.916,D**	G/A
754	PTPRQ	p.V645D	rs10778752	**0.917,D**	A/A

755	ZNF880	p.K471R	rs55748277	**0.917,D**	A/G
756	CPA4	p.A282T	rs570075096	**0.917,D**	G/A
757	OR51B2	p.L134F	rs10837814	**0.918,D**	G/A
758	KLLN	p.R128G	rs201652303	**0.918,D**	G/C
759	ZNF404	p.R474C	rs76311065	**0.918,D**	G/A
760	KIAA2013	p.S667F	rs11555351	**0.919,D**	G/A
761	CHI3L2	p.A182V	rs11556868	**0.919,D**	C/T
762	C11orf24	p.G97V	rs3802746	**0.919,D**	A/A
763	SMG6	p.R291P	rs1885986	**0.921,D**	C/G
764	VASN	p.G551R	rs367830549	**0.921,D**	G/A
765	NPAS4	p.G676V	rs1371192899	**0.922,D**	G/T
766	HLA-DQB1	p.R87P	rs1130380	**0.922,D**	G/G
767	KRTAP10-1	p.V101M	rs233319	**0.922,D**	C/T
768	MACC1	p.R804T	rs3735615	**0.922,D**	G/G
769	IL11	p.R112H	rs4252548	**0.922,D**	C/T
770	ASMTL	p.S228P	rs11553051	**0.923,D**	A/G
771	MYO19	p.N176S	rs2306595	**0.923,D**	T/C
772	ZFP28	p.S141W	rs34136271	**0.923,D**	C/G
773	NVL	p.V404I	rs34631151	**0.923,D**	C/T
774	GRIN3B	p.T157M	rs2240154	**0.925,D**	C/T
775	NOL9	p.R10W	rs4908923	**0.925,D**	A/A
776	MAVS	p.S409F	rs7269320	**0.925,D**	C/T
777	PON1	p.L55M	rs854560	**0.925,D**	A/T
778	INADL	p.E362A	rs1286823	**0.926,D**	A/C
779	FBXO2	p.K118T	rs9614	**0.926,D**	T/G
780	MAGEF1	p.E236A	rs9872799	**0.926,D**	G/G
781	NAALADL2	p.P622R	rs9866564	**0.927,D**	G/G
782	KRT81	p.G52R	rs2071588	**0.928,D**	G/G
783	ITIH1	p.E585V	rs678	**0.928,D**	A/T
784	OR4K17	p.K159N	rs8005245	**0.928,D**	G/C
785	ZNF443	p.K480T	rs10402252	**0.930,D**	G/G
786	OR13C5	p.P79S	rs7025570	**0.931,D**	G/A
787	QRICH2	p.E1036Q	rs2279052	**0.932,D**	C/G
788	ASPM	p.S2562G	rs41310927	**0.932,D**	T/C
789	OR5H14	p.G64R	rs4241468	**0.933,D**	A/A
790	PCDHGA8	p.L16R	rs726684	**0.933,D**	T/G
791	KRT35	p.S36P	rs743686	**0.933,D**	A/G
792	CPZ	p.R19P	rs79736750	**0.933,D**	G/C
793	MICA	p.R29P	rs9380254	**0.933,D**	G/C
794	CDCP2	p.G244R	rs3766465	**0.934,D**	C/T
795	MUC16	p.L1833F	rs4520945	**0.934,D**	G/A
796	CELSR1	p.S664W	rs4823850	**0.935,D**	C/C
797	OR9Q2	p.C179R	rs34337292	**0.936,D**	T/C
798	SMYD4	p.P382R	rs3809875	**0.936,D**	G/C
799	KRT79	p.A393V	rs17688627	**0.937,D**	G/A

800	FARP1	p.H644Y	rs61730892	**0.937,D**	C/T
801	TPSG1	p.S138F	rs113856625	**0.939,D**	A/A
802	ISCU	p.A12V	rs2287555	**0.939,D**	T/T
803	HPS1	p.P167R	rs2296434	**0.939,D**	G/C
804	CAPN8	p.S245Y	rs35539373	**0.939,D**	G/T
805	PKD1L2	p.G129D	rs7185774	**0.939,D**	C/T
806	ACSM5	p.P352R	rs8062344	**0.940,D**	C/G
807	KAZN	p.E88D	N/A	**0.941,D**	A/C
808	MTHFR	p.A222V	rs1801133	**0.941,D**	G/A
809	PYGB	p.A303S	rs2228976	**0.941,D**	G/T
810	FCGR3A	p.L66H	rs10127939	**0.942,D**	A/T
811	TTC21B	p.V201M	rs1432273	**0.942,D**	T/T
812	ARFGAP2	p.S364F	N/A	**0.943,D**	G/A
813	RABL6	p.E382Q	rs2811741	**0.943,D**	G/C
814	FRG2C	p.D143G	rs79973298	**0.943,D**	A/G
815	HLA-DQB1	p.A118E	rs9274380	**0.943,D**	T/T
816	COL4A3	p.P574L	rs28381984	**0.944,D**	T/T
817	KRT3	p.R375G	rs3887954	**0.944,D**	G/C
818	SELE	p.S149R	rs5361	**0.944,D**	T/G
819	OR7A17	p.A237T	rs13345394	**0.945,D**	C/T
820	OR13F1	p.T254M	rs7030820	**0.947,D**	C/T
821	PKD1L2	p.P512L	rs7205673	**0.947,D**	A/A
822	COL17A1	p.T210M	rs805708	**0.947,D**	A/A
823	SYCP2L	p.P672S	rs1225746	**0.948,D**	T/T
824	LRRC15	p.P292L	rs13070515	**0.948,D**	G/A
825	EXPH5	p.R12G	rs2640738	**0.948,D**	T/C
826	SLC25A5	p.G121C	rs753913830	**0.948,D**	G/T
827	TNXB	p.P2412L	rs12524664	**0.949,D**	G/A
828	EFTUD1	p.I617V	rs1128431	**0.950,D**	T/C
829	USP21	p.G321D	rs17356051	**0.950,D**	G/A
830	EPYC	p.S150C	rs17784152	**0.950,D**	G/C
831	ITGAM	p.R246Q	rs199671976	**0.950,D**	G/A
832	PLXND1	p.L1412V	rs2625973	**0.950,D**	A/C
833	ANXA9	p.D166G	rs267733	**0.950,D**	A/G
834	RD3	p.G57V	rs767481165	**0.950,D**	C/A
835	MYO18A	p.A977V	rs8076604	**0.950,D**	G/A
836	HLA-DRB1	p.K41T	rs1136756	**0.951,D**	T/G
837	MRPS31	p.D279N	rs13508	**0.951,D**	C/T
838	AARS	p.G931S	rs149377346	**0.951,D**	C/T
839	AP5B1	p.L211F	rs12146493	**0.952,D**	A/A
840	CD5	p.A471V	rs2229177	**0.952,D**	C/T
841	ZIM3	p.I379V	rs4801433	**0.952,D**	T/C
842	AHNAK2	p.A2015P	rs117379881	**0.953,D**	G/G
843	WFS1	p.R456H	rs1801208	**0.953,D**	G/A
844	MATN2	p.T187M	rs2290472	**0.953,D**	C/T

845	ZBBX	p.K160N	rs4619784	**0.953,D**	T/A
846	RGPD4	p.W1646R	rs832352	**0.953,D**	T/A
847	RBMX	p.S337N	rs35899675	**0.954,D**	C/T
848	ERV3-1	p.C192Y	rs34639489	**0.955,D**	C/T
849	BPIFA3	p.A41E	rs17124391	**0.956,D**	C/A
850	TPO	p.S398T	rs2175977	**0.956,D**	G/C
851	MYOC	p.R76K	rs2234926	**0.956,D**	C/T
852	KIF24	p.S837F	rs41274041	**0.956,D**	G/A
853	NFS1	p.R145W	rs148627243	**0.957,D**	G/A
854	CCDC86	p.Q153H	rs2074421	**0.957,D**	C/C
855	PFAS	p.R811W	rs147698247	**0.958,D**	C/T
856	CYP2D6	p.L213P	rs3021082	**0.958,D**	A/G
857	ANKDD1A	p.K355E	rs34988193	**0.958,D**	A/G
858	CCDC116	p.R122W	rs861853	**0.958,D**	C/T
859	ITGB4	p.L1779P	rs871443	**0.958,D**	T/C
860	ZC3HC1	p.R342H	rs11556924	**0.959,D**	C/T
861	THAP7	p.E69Q	rs145388226	**0.959,D**	C/G
862	TSPAN32	p.R164C	rs148601311	**0.959,D**	C/T
863	IDI1	p.G12A	rs4880760	**0.959,D**	C/G
864	FFAR3	p.Y249C	rs763450506	**0.959,D**	A/G
865	PLCE1	p.R548L	rs17417407	**0.960,D**	G/T
866	NEMF	p.S257C	rs3100906	**0.960,D**	A/A
867	SRBD1	p.K811R	rs3755072	**0.960,D**	T/C
868	ALPK2	p.P1449S	rs3809982	**0.960,D**	A/A
869	SLX4	p.S1271F	rs3810813	**0.960,D**	G/A
870	MOV10L1	p.R182C	rs3810971	**0.960,D**	C/T
871	FAM169A	p.E511K	rs76455982	**0.960,D**	C/T
872	OR52I2	p.T167M	rs1847632	**0.961,D**	C/T
873	USP16	p.Q141H	rs2274802	**0.961,D**	A/T
874	EXO1	p.N279S	rs4149909	**0.961,D**	A/G
875	ACSM1	p.A176P	rs61740631	**0.961,D**	C/G
876	SPECC1	p.S274R	rs9908032	**0.961,D**	C/G
877	ACACB	p.D1481V	rs113524436	**0.962,D**	A/T
878	SLC45A2	p.L374F	rs16891982	**0.962,D**	C/G
879	HLA-DRB5	p.K41T	rs200581589	**0.962,D**	T/G
880	OR52N4	p.Y170S	rs75108386	**0.962,D**	A/C
881	CCDC22	p.R384C	rs143790434	**0.963,D**	T/T
882	ARHGEF37	p.P489L	rs9324624	**0.963,D**	T/T
883	TRAPPC5	p.R7C	rs753176463	**0.964,D**	C/T
884	C6orf118	p.G271E	rs17852379	**0.964,D**	C/T
885	CNOT1	p.R299Q	rs34830321	**0.964,D**	C/T
886	TRIM58	p.T374M	rs3811444	**0.964,D**	T/T
887	FAM186A	p.M2193I	rs6580742	**0.964,D**	C/T
888	OR6C68	p.F110I	rs7304753	**0.964,D**	T/A
889	CROCC	p.D586H	rs9435714	**0.964,D**	G/C

890	ZNF286B	p.T490S	rs9912644	0.964,D	G/C
891	FSIP1	p.G528A	rs16969386	0.965,D	C/G
892	LAMA2	p.A2587V	rs2229848	0.965,D	T/T
893	P3H3	p.R304C	rs35359746	0.965,D	C/T
894	RNF213	p.L4649V	rs61745599	0.965,D	C/G
895	HJURP	p.E723G	rs10511	0.966,D	T/C
896	FKBP9	p.V367M	rs150348129	0.966,D	G/A
897	MUC16	p.S5885F	rs1862458	0.966,D	G/A
898	LKAAEAR1	p.G9W	rs4431000	0.966,D	A/A
899	NPSR1	p.S241R	rs727162	0.966,D	C/G
900	TRPM1	p.A486V	rs751743414	0.966,D	G/A
901	ZNF764	p.A332V	rs17850402	0.967,D	G/A
902	ADAMTSL4	p.R87P	rs199599791	0.968,D	G/C
903	LRRC43	p.Q71K	rs11060094	0.968,D	C/A
904	CACTIN	p.S108L	rs55862054	0.968,D	G/A
905	ZNF764	p.R52Q	rs61744716	0.968,D	C/T
906	MYH15	p.H504Y	rs9868484	0.968,D	A/A
907	ZNF714	p.K332R	rs2884554	0.969,D	G/G
908	CES1	p.G18V	rs3826190	0.969,D	C/A
909	RBMX	p.Y357H	rs76876438	0.969,D	A/G
910	RBMX	p.R324P	rs77794331	0.969,D	C/G
911	IQGAP3	p.V410L	rs11264498	0.970,D	G/G
912	HK3	p.G246D	rs61749653	0.970,D	C/T
913	SLF2	p.S541Y	rs10883563	0.971,D	A/A
914	BZRAP1	p.A645G	rs61739511	0.971,D	G/C
915	HMCN1	p.G1292V	N/A	0.972,D	G/T
916	OR52W1	p.L254Q	rs11040799	0.972,D	A/A
917	COL6A5	p.T1280P	rs12488457	0.972,D	A/C
918	ZNF676	p.G27E	rs8104929	0.972,D	T/T
919	PKHD1L1	p.Y440H	rs964307	0.972,D	T/C
920	HLA-A	p.A270S	rs1059632	0.973,D	G/T
921	IPO4	p.P580A	rs11550452	0.973,D	G/C
922	WDR46	p.V341A	rs14398	0.973,D	A/G
923	CCT6B	p.V48A	rs2230552	0.973,D	A/G
924	KRTAP10-1	p.R241Q	rs233317	0.973,D	C/T
925	TYR	p.S192Y	rs1042602	0.974,D	C/A
926	OR2D3	p.L82I	rs10839658	0.974,D	C/A
927	NIPAL1	p.I324V	rs13116684	0.974,D	A/G
928	C10orf71	p.D45G	rs151171891	0.974,D	A/G
929	HLA-A	p.S14L	rs2230954	0.974,D	C/T
930	R3HDM1	p.Q632P	rs2305165	0.974,D	A/C
931	PRX	p.P1083R	rs3745202	0.974,D	G/C
932	KIAA0430	p.Y223H	rs775204838	0.974,D	A/G
933	BAG3	p.I94F	rs145393807	0.975,D	A/T
934	OR3A1	p.R125Q	rs703903	0.975,D	C/T

935	C7orf57	p.P201H	rs2708890	**0.976,D**	A/A
936	OR2T8	p.A231G	rs4595394	**0.976,D**	G/G
937	OR10H5	p.L216P	rs62106066	**0.976,D**	T/C
938	SCFD2	p.L512S	rs7675987	**0.976,D**	A/G
939	KRT26	p.W118R	rs9898164	**0.976,D**	A/G
940	C9orf43	p.P271A	rs111748634	**0.977,D**	C/G
941	EPPK1	p.R1066C	rs116925616	**0.977,D**	G/A
942	EPPK1	p.R1066H	rs118079568	**0.977,D**	C/T
943	RFPL2	p.R341C	rs136468	**0.977,D**	A/A
944	MAP1S	p.S411C	rs17710707	**0.977,D**	C/G
945	KRT32	p.S222Y	rs2071561	**0.977,D**	T/T
946	ACTN3	p.E678A	rs2229456	**0.977,D**	A/C
947	IPPK	p.L376F	rs2277170	**0.977,D**	G/A
948	TIMELESS	p.P1018L	rs2291739	**0.977,D**	G/A
949	APOL4	p.A316E	rs6000173	**0.977,D**	G/T
950	RGPD3	p.D111N	rs62152530	**0.977,D**	C/T
951	MCTP1	p.R612K	rs9885412	**0.977,D**	C/T
952	PKD1L1	p.E645D	N/A	**0.978,D**	T/A
953	C17orf74	p.S108A	rs13290	**0.978,D**	T/G
954	TAS2R42	p.Y175F	rs35969491	**0.978,D**	T/A
955	FSCN3	p.A24S	rs3779536	**0.978,D**	G/T
956	C1orf106	p.Y347F	rs41313912	**0.978,D**	A/T
957	DOCK8	p.P97T	rs529208	**0.978,D**	C/A
958	SCML4	p.R337W	rs56215636	**0.978,D**	G/A
959	NUP107	p.E394G	rs899872828	**0.978,D**	A/G
960	COL4A3	p.G43R	rs13424243	**0.979,D**	G/C
961	SLC25A5	p.R138H	rs200550329	**0.979,D**	G/A
962	OR1D5	p.P210L	rs2676567	**0.979,D**	G/A
963	ZNF117	p.K112N	rs3807068	**0.979,D**	T/G
964	FSCN2	p.R406P	rs750102960	**0.979,D**	G/C
965	OR1I1	p.Y252S	rs8105737	**0.979,D**	C/C
966	HPSE2	p.Y579F	rs10883100	**0.980,D**	T/A
967	KRT33B	p.E85K	rs12450621	**0.980,D**	C/T
968	CYP4F2	p.V433M	rs2108622	**0.980,D**	C/T
969	ADAMTS7	p.T307M	rs2127898	**0.980,D**	G/A
970	VWDE	p.C1050Y	rs35171886	**0.980,D**	C/T
971	SERPINB8	p.H359R	rs3826616	**0.980,D**	A/G
972	GATAD2A	p.R293H	rs748958687	**0.980,D**	G/A
973	NCAPG2	p.A132V	N/A	**0.981,D**	G/A
974	PSPH	p.R65H	rs200442078	**0.981,D**	C/T
975	LYZL2	p.K13R	rs74551644	**0.981,D**	T/C
976	IPPK	p.R130W	rs75957544	**0.981,D**	G/A
977	TYSND1	p.S239R	rs1166621779	**0.982,D**	T/G
978	COL6A2	p.R680H	rs1042917	**0.982,D**	G/A
979	DLAT	p.D451N	rs10891314	**0.982,D**	G/A

980	BTC	p.L124M	rs11938093	**0.982,D**	A/T
981	POM121L2	p.G644C	rs16897515	**0.982,D**	C/A
982	FRG2C	p.D9N	rs201766868	**0.982,D**	G/A
983	CTAGE9	p.L398V	rs202051647	**0.982,D**	G/C
984	FUT2	p.G258S	rs602662	**0.982,D**	A/A
985	PKP1	p.R705W	rs61818256	**0.982,D**	C/T
986	FGGY	p.T230M	rs62623594	**0.982,D**	C/T
987	MLX	p.Q169R	rs665268	**0.982,D**	A/G
988	SCFD2	p.G283V	rs79025139	**0.982,D**	C/A
989	MYOM1	p.E704K	rs149528866	**0.983,D**	C/T
990	SLC25A5	p.L173P	rs200606066	**0.983,D**	T/C
991	ECD	p.D667G	rs2271904	**0.983,D**	T/C
992	SVOPL	p.F385C	rs2305816	**0.983,D**	A/C
993	HMMR	p.R92C	rs299284	**0.983,D**	C/T
994	ASPM	p.L2647I	rs3762271	**0.983,D**	G/T
995	SAT2	p.R126C	rs13894	**0.984,D**	G/A
996	SLC25A5	p.G73S	rs143413528	**0.984,D**	G/A
997	PLA2G3	p.L157V	rs2074735	**0.984,D**	G/C
998	ATP10A	p.W1172C	rs2076742	**0.984,D**	C/G
999	RIOK2	p.S96C	rs2544773	**0.984,D**	C/C
1000	WDR72	p.P306L	rs551225	**0.984,D**	G/A
1001	OR10H5	p.C204G	rs61738869	**0.984,D**	T/G
1002	SVOPL	p.I362R	rs773196860	**0.984,D**	A/C
1003	KRT40	p.T153M	rs9908304	**0.984,D**	A/A
1004	ATXN7	p.K264R	rs1053338	**0.985,D**	A/G
1005	FLNB	p.D1157N	rs1131356	**0.985,D**	G/A
1006	ULK3	p.K328R	rs12898397	**0.985,D**	C/C
1007	SLC35G5	p.A43V	rs13260331	**0.985,D**	C/T
1008	RTP5	p.G101E	rs7420371	**0.985,D**	A/A
1009	WASHC2A	p.V1285M	rs1427726813	**0.986,D**	G/A
1010	OR2W3	p.E196D	rs12139390	**0.986,D**	C/C
1011	S100Z	p.E23A	rs1320308	**0.986,D**	C/C
1012	KRT39	p.L383M	rs17843023	**0.986,D**	G/T
1013	PDPR	p.G564S	rs369641860	**0.986,D**	G/A
1014	SCARB1	p.G2S	rs4238001	**0.986,D**	C/T
1015	EP400	p.P2792S	rs542350095	**0.986,D**	C/T
1016	TRPM3	p.R1542Q	rs6560142	**0.986,D**	C/T
1017	ST8SIA4	p.S221N	rs759794120	**0.986,D**	C/T
1018	HOPX	p.E110K	rs76451666	**0.986,D**	C/T
1019	LAMA5	p.H1717Y	rs875379	**0.986,D**	G/A
1020	OR5R1	p.A274V	rs998544	**0.986,D**	G/A
1021	NBPF26, NOTCH2NLI	p.T235S	rs1387085969	**0.987,D**	T/T
1022	C8B	p.E46K	rs12067507	**0.987,D**	C/T
1023	OR56B4	p.P277S	rs1462983	**0.987,D**	C/T
1024	SLC25A5	p.N77T	rs148294496	**0.987,D**	A/C

1025	PDCD4	p.M390K	rs201057391	**0.987,D**	T/A
1026	POLQ	p.A2547V	rs2306211	**0.987,D**	G/A
1027	OR14C36	p.D231Y	rs28545014	**0.987,D**	G/T
1028	ANO4	p.G115A	rs34162417	**0.987,D**	G/C
1029	PKD1L3	p.V903I	rs9921412	**0.987,D**	T/T
1030	PRAMEF10	p.K99I	rs3121398	**0.988,D**	T/A
1031	MB21D1	p.P261H	rs610913	**0.988,D**	T/T
1032	VKORC1	p.D36Y	rs61742245	**0.988,D**	C/A
1033	MPHOSPH8	p.D460Y	rs75390100	**0.988,D**	G/T
1034	OR9G9,OR9G1	p.V279E	rs79251113	**0.988,D**	T/A
1035	CNTNAP3	p.G714V	rs10974150	**0.989,D**	A/A
1036	PJA1	p.E418D	rs11539157	**0.989,D**	A/A
1037	TMC6	p.L153F	rs12449858	**0.989,D**	G/A
1038	PMFBP1	p.E48K	rs35370634	**0.989,D**	C/T
1039	TSPAN8	p.G73A	rs3763978	**0.989,D**	C/G
1040	OR2G2	p.L167P	rs10925085	**0.990,D**	T/C
1041	CLDN3	p.P134L	rs139191328	**0.990,D**	G/A
1042	APBA2	p.S55N	rs142678624	**0.990,D**	G/A
1043	CUBN	p.N2157D	rs144360241	**0.990,D**	T/C
1044	MTRR	p.I49M	rs1801394	**0.990,D**	G/G
1045	IL17F	p.E126G	rs2397084	**0.990,D**	T/C
1046	FNDC3B	p.A452S	rs35409041	**0.990,D**	G/T
1047	OR52L1	p.D88N	rs4501959	**0.990,D**	C/T
1048	ADGB	p.E1621D	rs1052444	**0.991,D**	A/T
1049	CROCC	p.D463Y	rs114323849	**0.991,D**	G/T
1050	VWA5B1	p.R880H	rs11582960	**0.991,D**	A/A
1051	PRDM7	p.D90E	rs12925933	**0.991,D**	A/C
1052	FBXO39	p.L231F	rs1509123	**0.991,D**	C/T
1053	HS6ST1	p.D87E	rs200979099	**0.991,D**	G/T
1054	HLA-DRB1	p.L28F	rs201614260	**0.991,D**	C/G
1055	OR51F2	p.D84G	rs35003053	**0.991,D**	A/G
1056	LOXHD1	p.G632C	rs35088381	**0.991,D**	C/A
1057	ZMIZ2	p.L408F	rs3735478	**0.991,D**	G/T
1058	RRS1	p.R191L	rs3739336	**0.991,D**	G/T
1059	PLEKHH1	p.Q113L	rs3825723	**0.991,D**	A/T
1060	OR5H6	p.C195R	rs9853887	**0.991,D**	C/C
1061	CCHCR1	p.L179Q	rs11540822	**0.992,D**	A/T
1062	OR8D4	p.L55R	rs17127947	**0.992,D**	T/G
1063	STON2	p.S851A	rs2241621	**0.992,D**	C/C
1064	BCAS4	p.E56D	rs2272962	**0.992,D**	G/T
1065	DOCK8	p.D63N	rs3209441	**0.992,D**	G/A
1066	LRP2	p.I4210L	rs4667591	**0.992,D**	G/G
1067	FAM81B	p.R134C	rs76962324	**0.992,D**	C/T
1068	ZNF703	p.P307S	rs79707182	**0.992,D**	C/T
1069	MCPH1	p.D314H	rs930557	**0.992,D**	C/C

1070	MYH13	p.R1438C	rs141961168	**0.993,D**	G/A
1071	PLEKHG7	p.D99G	rs17790310	**0.993,D**	A/G
1072	PEX6	p.A721V	rs35830695	**0.993,D**	G/A
1073	FAM198A	p.G539S	rs3732858	**0.993,D**	G/A
1074	UGT2A1	p.L197S	rs41292307	**0.993,D**	A/G
1075	SYT8	p.T303M	rs484955	**0.993,D**	T/T
1076	MUC6	p.Y1826D	rs55903826	**0.993,D**	A/C
1077	CYP4F8	p.T152M	rs61746468	**0.993,D**	C/T
1078	PLEKHA6	p.A128T	rs61755454	**0.993,D**	C/T
1079	C6orf222	p.L582V	rs743852	**0.993,D**	G/C
1080	FBLN5	p.V361I	rs757825263	**0.993,D**	C/T
1081	HLA-DRB1	p.A169T	rs78916069	**0.993,D**	C/T
1082	OR10A2	p.K258T	rs7926083	**0.993,D**	A/C
1083	VRK2	p.I167V	rs1051061	**0.994,D**	A/G
1084	SPATA6	p.C478Y	rs1056042	**0.994,D**	C/T
1085	ARSB	p.V358M	rs1065757	**0.994,D**	T/T
1086	TRIM66	p.H324R	rs11042023	**0.994,D**	C/C
1087	TYR	p.R402Q	rs1126809	**0.994,D**	G/A
1088	SAA1	p.V75A	rs1136747	**0.994,D**	T/C
1089	FAM179A	p.Q265R	rs12623297	**0.994,D**	A/G
1090	ZBBX	p.A473G	rs13096767	**0.994,D**	G/C
1091	MRI1	p.T209R	rs141252695	**0.994,D**	C/G
1092	EEF2K	p.P75A	rs17841292	**0.994,D**	C/G
1093	DMD	p.R1622H	rs1801187	**0.994,D**	T/T
1094	SPATA19	p.A50V	rs2282602	**0.994,D**	G/A
1095	PKD1L1	p.V312F	rs2686817	**0.994,D**	A/A
1096	MUC6	p.P1873Q	rs34844844	**0.994,D**	G/T
1097	OR5I1	p.L50S	rs4367963	**0.994,D**	A/G
1098	OR9G4	p.V222A	rs513873	**0.994,D**	A/G
1099	MAP2K3	p.R67W	rs56216806	**0.994,D**	C/T
1100	ABCB5	p.G810V	rs62453384	**0.994,D**	G/T
1101	RUNX3	p.I18N	rs6672420	**0.994,D**	A/T
1102	FER1L5	p.R1523Q	rs745681758	**0.994,D**	G/A
1103	FRMD4B	p.S947L	rs9831516	**0.994,D**	A/A
1104	KIAA0753	p.E375D	rs9889363	**0.994,D**	A/A
1105	NEB	p.W3603C	rs10172023	**0.995,D**	C/G
1106	HLA-B	p.E70A	rs1050538	**0.995,D**	T/G
1107	GUCA1C	p.G148D	rs10933973	**0.995,D**	C/A
1108	EVC	p.R576Q	rs1383180	**0.995,D**	G/A
1109	TMF1	p.D798H	rs1532918	**0.995,D**	C/G
1110	SPTB	p.G1408R	rs17245552	**0.995,D**	C/G
1111	STEAP2	p.F17C	rs194520	**0.995,D**	T/G
1112	MUC6	p.L1836H	rs200241162	**0.995,D**	A/T
1113	TAS2R19	p.A227P	rs200715232	**0.995,D**	C/G
1114	AHNAK2	p.K2170R	rs201127689	**0.995,D**	C/C

1115	ABCA9	p.K1306T	rs2302294	**0.995,D**	G/G
1116	GSDMB	p.G304R	rs2305479	**0.995,D**	C/T
1117	KRTAP10-1	p.P39L	rs233320	**0.995,D**	G/A
1118	PLET1	p.S142P	rs2564872	**0.995,D**	A/G
1119	INMT	p.F254C	rs4720015	**0.995,D**	G/G
1120	ADGRV1	p.P1987L	rs4916685	**0.995,D**	T/T
1121	DCK	p.P122S	rs67437265	**0.995,D**	C/T
1122	MS4A15	p.L47R	rs1032939	**0.996,D**	T/G
1123	PKD1L3	p.S1176R	rs1035543	**0.996,D**	C/C
1124	ZNF683	p.D48G	rs10794532	**0.996,D**	C/C
1125	PDHX	p.T101A	rs11539202	**0.996,D**	A/G
1126	RELN	p.S630R	rs115734214	**0.996,D**	T/G
1127	CD300C	p.T71I	rs11870245	**0.996,D**	G/A
1128	CLEC12B	p.T6N	rs1359082	**0.996,D**	C/A
1129	CATSPERD	p.E596D	rs143660393	**0.996,D**	G/C
1130	SVEP1	p.E3412Q	rs145193955	**0.996,D**	C/G
1131	HPSE2	p.Y211H	rs147866530	**0.996,D**	A/G
1132	HLA-DRB1	p.P25R	rs148093782	**0.996,D**	G/C
1133	RNF40	p.L261F	rs150097902	**0.996,D**	C/T
1134	PMFBP1	p.K768N	rs16973716	**0.996,D**	T/G
1135	TCP10L2	p.G254R	rs200019718	**0.996,D**	G/A
1136	MUC4	p.A4101S	rs2246901	**0.996,D**	A/A
1137	C1orf131	p.L28V	rs2274067	**0.996,D**	G/C
1138	CHD1L	p.S671C	rs2275249	**0.996,D**	C/G
1139	TTN	p.R19479H	rs2288569	**0.996,D**	C/T
1140	ITSN2	p.A1520T	rs2303291	**0.996,D**	C/T
1141	GP6	p.P314A	rs2304166	**0.996,D**	C/C
1142	SNTG2	p.S168Y	rs28505970	**0.996,D**	C/A
1143	ZAN	p.N2073S	rs314300	**0.996,D**	A/G
1144	POLI	p.F532S	rs3218786	**0.996,D**	T/C
1145	SHROOM3	p.P469A	rs344141	**0.996,D**	G/G
1146	MUC6	p.T1854M	rs34507884	**0.996,D**	G/A
1147	OR51G1	p.R124H	rs34742470	**0.996,D**	C/T
1148	TTC21A	p.R622W	rs35581078	**0.996,D**	C/T
1149	GPR45	p.L312F	rs35946826	**0.996,D**	C/T
1150	APLF	p.Q433K	rs36021078	**0.996,D**	C/A
1151	A2ML1	p.I1054T	rs371117844	**0.996,D**	T/C
1152	SYNPO2L	p.P707L	rs3812629	**0.996,D**	G/A
1153	TTN	p.R34594H	rs3829747	**0.996,D**	C/T
1154	OR1N2	p.F194L	rs41297203	**0.996,D**	T/C
1155	SLCO1B1	p.V174A	rs4149056	**0.996,D**	T/C
1156	FRMD7	p.S281L	rs5977625	**0.996,D**	A/A
1157	INCENP	p.E644D	rs7129085	**0.996,D**	G/T
1158	RBMX	p.G379R	rs72615484	**0.996,D**	C/G
1159	KCNMB3	p.A31T	rs7645550	**0.996,D**	C/T

1160	UNC45A	p.T781M	rs8041035	**0.996,D**	C/T
1161	OGG1	p.N151I	rs764308935	**0.997,D**	A/T
1162	USP19	p.V956A	N/A	**0.997,D**	A/G
1163	MICA	p.L48V	rs1051790	**0.997,D**	C/G
1164	BCAS1	p.S583P	rs1055246	**0.997,D**	A/G
1165	UNC45B	p.I850N	rs11654824	**0.997,D**	T/A
1166	HLA-DRB5	p.D31G	rs139583918	**0.997,D**	T/C
1167	OR2AG2	p.G233R	rs143018775	**0.997,D**	C/T
1168	FAM65B	p.W15R	rs148823241	**0.997,D**	A/G
1169	PCDHB7	p.V689L	rs1811237	**0.997,D**	G/T
1170	SLC25A5	p.G183V	rs200974395	**0.997,D**	G/T
1171	AKR7L	p.C186Y	rs2235794	**0.997,D**	T/T
1172	CHIA	p.F354S	rs2275254	**0.997,D**	T/C
1173	TRERF1	p.C834S	rs2295275	**0.997,D**	A/T
1174	DPYD	p.M166V	rs2297595	**0.997,D**	T/C
1175	ADGRV1	p.N2345S	rs2366926	**0.997,D**	G/G
1176	ADAMTS13	p.P618A	rs28647808	**0.997,D**	C/G
1177	SLC7A13	p.V249M	rs2976189	**0.997,D**	T/T
1178	DNAH10	p.T1986M	rs33935373	**0.997,D**	C/T
1179	NPHS1	p.R408Q	rs33950747	**0.997,D**	C/T
1180	THEG	p.R193C	rs35119230	**0.997,D**	A/A
1181	AHSG	p.R317C	rs35457250	**0.997,D**	C/T
1182	ZCCHC14	p.V693M	rs3748400	**0.997,D**	C/T
1183	LGALS3	p.P64H	rs4644	**0.997,D**	C/A
1184	AGT	p.T207M	rs4762	**0.997,D**	G/A
1185	MATN3	p.E252K	rs52826764	**0.997,D**	C/T
1186	OBSCN	p.G4209R	rs56218706	**0.997,D**	G/A
1187	OBSL1	p.R1767Q	rs59332477	**0.997,D**	T/T
1188	SVEP1	p.R229G	rs61751937	**0.997,D**	G/C
1189	GALNTL5	p.C124R	rs6960270	**0.997,D**	C/C
1190	FAM131C	p.R107Q	rs71510977	**0.997,D**	C/T
1191	HLA-DRB5	p.L28F	rs72508462	**0.997,D**	C/G
1192	MUC6	p.T1889I	rs747429892	**0.997,D**	G/A
1193	HLA-C	p.R155S	rs76907552	**0.997,D**	G/T
1194	HSPG2	p.A1503V	rs897471	**0.997,D**	A/A
1195	GEMIN4	p.A579G	rs910925	**0.997,D**	G/C
1196	ANXA11	p.R230C	rs1049550	**0.998,D**	G/A
1197	SPAG17	p.P1348L	rs10923472	**0.998,D**	G/A
1198	TNXB	p.R2704H	rs10947230	**0.998,D**	C/T
1199	OR51I2	p.R263H	rs11037502	**0.998,D**	G/A
1200	SPATA3	p.C122S	rs12105962	**0.998,D**	G/C
1201	CCHCR1	p.R627Q	rs130072	**0.998,D**	C/T
1202	NEB	p.K2613N	rs13013209	**0.998,D**	C/G
1203	TAS2R31	p.D45H	rs143614038	**0.998,D**	C/G
1204	UGT2A2,UGT2A1	p.S488Y	rs144056072	**0.998,D**	G/T

1205	DOPEY2	p.R2055C	rs145488940	**0.998,D**	C/T
1206	PSG2	p.G118R	rs149579909	**0.998,D**	C/G
1207	VWDE	p.C890F	rs17165906	**0.998,D**	C/A
1208	SPAG17	p.E158V	rs17185492	**0.998,D**	T/A
1209	NBPF3	p.Y58C	rs1827293	**0.998,D**	G/G
1210	KRT78	p.L92P	rs2013335	**0.998,D**	A/G
1211	C5orf46	p.S4L	rs2250145	**0.998,D**	A/A
1212	COLEC12	p.G606S	rs2305027	**0.998,D**	C/T
1213	HYDIN	p.E2306G	rs2502726	**0.998,D**	T/C
1214	KMT2C	p.C988F	rs28522267	**0.998,D**	C/A
1215	TRPM5	p.V335L	rs34350821	**0.998,D**	C/A
1216	ELSPBP1	p.P185L	rs35700000	**0.998,D**	C/T
1217	IGF2R	p.R292Q	rs370150079	**0.998,D**	G/A
1218	ANLN	p.S65W	rs3735400	**0.998,D**	C/G
1219	NEK11	p.E530V	rs3738000	**0.998,D**	A/T
1220	IRAK2	p.L392V	rs3844283	**0.998,D**	C/G
1221	DNHD1	p.L846M	rs4282961	**0.998,D**	C/A
1222	NWD2	p.L569M	rs4634233	**0.998,D**	A/A
1223	OR8B2	p.S273F	rs503220	**0.998,D**	A/A
1224	OBP2A	p.G130A	rs55695858	**0.998,D**	G/C
1225	ABCB5	p.D370G	rs61732039	**0.998,D**	A/G
1226	CRAMP1	p.P1084S	rs61746451	**0.998,D**	C/T
1227	APOB	p.A618V	rs679899	**0.998,D**	G/A
1228	FADS6	p.V256I	rs7219093	**0.998,D**	C/T
1229	AFAP1L2	p.L384F	rs73365341	**0.998,D**	G/A
1230	OR51I2	p.R263C	rs75620804	**0.998,D**	C/T
1231	TAS2R19	p.L235R	rs763119571	**0.998,D**	A/C
1232	AHNAK2	p.R51W	rs76650680	**0.998,D**	G/A
1233	OR10X1	p.T90M	rs77690058	**0.998,D**	G/A
1234	BMS1	p.R939G	rs779750824	**0.998,D**	C/G
1235	OR8B2	p.L164F	rs886202	**0.998,D**	A/A
1236	PKD1L3	p.V593M	rs9925415	**0.998,D**	C/T
1237	ESPNL	p.L866Q	rs1225875400	**0.999,D**	T/A
1238	ITGB1	p.I782F	N/A	**0.999,D**	T/A
1239	TMEM176A	p.L187F	rs10378	**0.999,D**	G/T
1240	NUDT6	p.R40Q	rs1048201	**0.999,D**	C/T
1241	OR4X1	p.P282S	rs10838852	**0.999,D**	T/T
1242	SPATS1	p.G8R	rs10948132	**0.999,D**	G/A
1243	ZAN	p.G1674C	rs10953303	**0.999,D**	G/T
1244	RBMXL1	p.G71A	rs111779380	**0.999,D**	C/G
1245	IL23R	p.R381Q	rs11209026	**0.999,D**	G/A
1246	OR51V1	p.G96W	rs11512276	**0.999,D**	C/G
1247	AVPI1	p.L36R	rs11556392	**0.999,D**	A/C
1248	CDC25C	p.G297R	rs11567997	**0.999,D**	C/G
1249	SLCO1A2	p.E172D	rs11568563	**0.999,D**	T/G

1250	SLC22A10	p.P516L	rs1201559	**0.999,D**	T/T
1251	SPINK6	p.P36T	rs12186491	**0.999,D**	A/A
1252	LARP1B	p.P462R	rs12508837	**0.999,D**	C/G
1253	EPPK1	p.Y744C	rs12543389	**0.999,D**	T/C
1254	OR2G2	p.P24A	rs12737801	**0.999,D**	C/G
1255	FSIP1	p.L411F	rs12908846	**0.999,D**	G/A
1256	ZNF404	p.H230Y	rs12977303	**0.999,D**	G/A
1257	ESYT2	p.C182S	rs13233513	**0.999,D**	A/T
1258	KIR3DS1,KIR3DL1	p.S107L	rs143159382	**0.999,D**	C/T
1259	SERPINB11	p.W188R	rs1506419	**0.999,D**	T/A
1260	KRTAP3-2	p.P53L	rs150830376	**0.999,D**	G/A
1261	A2ML1	p.C970Y	rs1558526	**0.999,D**	G/A
1262	GPR151	p.P40L	rs17104742	**0.999,D**	G/A
1263	STPG2	p.Y125H	rs17558193	**0.999,D**	A/G
1264	PRR16	p.P40T	rs17853861	**0.999,D**	C/A
1265	MBL2	p.G54D	rs1800450	**0.999,D**	T/T
1266	NQO1	p.P187S	rs1800566	**0.999,D**	G/A
1267	ETFA	p.T171I	rs1801591	**0.999,D**	G/A
1268	HPS4	p.H624Y	rs1894706	**0.999,D**	A/A
1269	SLC22A24	p.T58S	rs1939748	**0.999,D**	C/C
1270	MUC6	p.P1841T	rs199539548	**0.999,D**	G/T
1271	SNED1	p.S270Y	rs201554730	**0.999,D**	C/A
1272	TG	p.G653D	rs2069548	**0.999,D**	G/A
1273	OR1A2	p.G256C	rs2241091	**0.999,D**	G/T
1274	PARP4	p.A899T	rs2275660	**0.999,D**	C/T
1275	THSD7A	p.D771E	rs2285744	**0.999,D**	G/C
1276	CHIT1	p.G102S	rs2297950	**0.999,D**	C/T
1277	ACACB	p.A651T	rs2300455	**0.999,D**	G/A
1278	SLC22A14	p.P560R	rs240033	**0.999,D**	C/G
1279	ADAMTS5	p.R614H	rs2830585	**0.999,D**	C/T
1280	STKLD1	p.G535C	rs33919837	**0.999,D**	G/T
1281	FBN3	p.G1614S	rs33967815	**0.999,D**	C/T
1282	TRAF3IP2	p.D19N	rs33980500	**0.999,D**	C/T
1283	PPP1R26	p.R381L	rs34151777	**0.999,D**	G/T
1284	GTPBP10	p.L164F	rs35001814	**0.999,D**	G/C
1285	OR51G1	p.E113K	rs35264256	**0.999,D**	C/T
1286	PVRL4	p.P104T	rs35434391	**0.999,D**	G/T
1287	TTN	p.R32538H	rs3731749	**0.999,D**	C/T
1288	HEATR5A	p.V886M	rs3736918	**0.999,D**	T/T
1289	ZNF45	p.P303R	rs388685	**0.999,D**	G/C
1290	HIVEP3	p.P407T	rs41269477	**0.999,D**	G/T
1291	INPP5B	p.D871H	rs41311191	**0.999,D**	C/G
1292	MYH15	p.R454Q	rs4299484	**0.999,D**	C/T
1293	CYBA	p.Y72H	rs4673	**0.999,D**	A/G
1294	KRTAP10-11	p.S130F	rs4818952	**0.999,D**	C/T

1295	OR1L6	p.C150Y	rs4838012	**0.999,D**	G/A
1296	RCBTB1	p.A24V	rs4942848	**0.999,D**	G/A
1297	OR9G9,OR9G1	p.Y112C	rs4990194	**0.999,D**	A/G
1298	OR51B5	p.R88G	rs57900141	**0.999,D**	T/C
1299	SLC9B1	p.A473V	rs58374767	**0.999,D**	G/A
1300	OR51L1	p.N241Y	rs61729748	**0.999,D**	A/T
1301	NME8	p.I338T	rs62001870	**0.999,D**	T/C
1302	TSEN54	p.P483A	rs62088470	**0.999,D**	C/G
1303	CEP120	p.L602V	rs6595440	**0.999,D**	G/C
1304	DISC1	p.L639F	rs6675281	**0.999,D**	C/T
1305	FAM171A2	p.P129L	rs71371993	**0.999,D**	G/A
1306	CAPN8	p.A136V	rs71644745	**0.999,D**	G/A
1307	OR56B1	p.C106R	rs7397032	**0.999,D**	C/C
1308	CCDC66	p.R460Q	rs7637449	**0.999,D**	G/A
1309	DLGAP2	p.T375M	rs767514905	**0.999,D**	C/T
1310	USP45	p.K67E	rs7744845	**0.999,D**	T/C
1311	TTN	p.Y15591H	rs775496863	**0.999,D**	A/G
1312	ANKLE1	p.R548Q	rs77683348	**0.999,D**	G/A
1313	SUSD2	p.G70V	rs79232038	**0.999,D**	G/T
1314	RTN3	p.D501H	rs7936660	**0.999,D**	G/C
1315	OR5AN1	p.L289F	rs7941190	**0.999,D**	C/C
1316	ASPSCR1	p.L252Q	rs8074498	**0.999,D**	A/A
1317	SYNGR4	p.R27W	rs919804	**0.999,D**	C/T
1318	COL12A1	p.G3058S	rs970547	**0.999,D**	T/T
1319	LEXM	p.G126C	rs9782980	**0.999,D**	G/T
1320	UBASH3B	p.Y546C	rs1428664827	**1.000,D**	A/G
1321	IDO2	p.R248W	rs10109853	**1.000,D**	T/T
1322	OR6B2	p.C179R	rs10187574	**1.000,D**	A/G
1323	RAD17	p.L546R	rs1045051	**1.000,D**	T/G
1324	PIGC	p.P266S	rs1063412	**1.000,D**	A/A
1325	NXPE1	p.G211R	rs10891692	**1.000,D**	C/T
1326	SPATA31A6	p.P420L	rs11261835	**1.000,D**	T/T
1327	IGSF10	p.D2614N	rs112889898	**1.000,D**	C/T
1328	GAK	p.D1297N	rs1134921	**1.000,D**	C/T
1329	CFAP69	p.T867M	rs1134956	**1.000,D**	C/T
1330	HLA-A	p.G131W	rs1136702	**1.000,D**	G/T
1331	TBC1D26	p.A206V	rs11650318	**1.000,D**	T/T
1332	PXDNL	p.R781G	rs11992240	**1.000,D**	G/C
1333	INADL	p.G1543V	rs12140153	**1.000,D**	G/T
1334	NFXL1	p.P246L	rs12651301	**1.000,D**	G/A
1335	ERICH3	p.G319R	rs140864337	**1.000,D**	C/G
1336	ATP2C2	p.G809W	rs141229929	**1.000,D**	G/T
1337	SCN11A	p.L1158P	rs141686175	**1.000,D**	A/G
1338	SCML4	p.G158D	rs142985964	**1.000,D**	C/T
1339	PDHA2	p.R286P	rs147966234	**1.000,D**	G/C

1340	FAT4	p.G3524D	rs1567047	**1.000,D**	A/A
1341	FANK1	p.P12L	rs17153879	**1.000,D**	C/T
1342	QSOX1	p.G200A	rs17855475	**1.000,D**	G/C
1343	CFTR	p.R668C	rs1800100	**1.000,D**	C/T
1344	LOXL4	p.D405A	rs1983864	**1.000,D**	T/G
1345	MUSK	p.A763T	rs199507468	**1.000,D**	G/A
1346	PCDHGA11	p.G469C	rs199543811	**1.000,D**	G/T
1347	FER1L6	p.G943W	rs200894396	**1.000,D**	G/T
1348	RHBG	p.G46D	rs2245623	**1.000,D**	A/A
1349	CYP4B1	p.R376C	rs2297809	**1.000,D**	C/T
1350	SMPDL3A	p.P161S	rs28385609	**1.000,D**	C/T
1351	NDUFA9	p.P294L	rs34076756	**1.000,D**	C/T
1352	GMPR2	p.G206D	rs34354104	**1.000,D**	G/A
1353	LOXHD1	p.G626C	rs34589386	**1.000,D**	C/A
1354	PRSS48	p.C53Y	rs36097019	**1.000,D**	A/A
1355	PCDHA1	p.N449H	rs3733712	**1.000,D**	A/C
1356	TLR3	p.L412F	rs3775291	**1.000,D**	C/T
1357	BCL2A1	p.G82D	rs3826007	**1.000,D**	C/T
1358	CYP2A7	p.R311C	rs3869579	**1.000,D**	A/A
1359	ACTL7A	p.G214S	rs41278347	**1.000,D**	G/A
1360	OR1D2	p.T240I	rs4300683	**1.000,D**	G/A
1361	ZNF221	p.C256R	rs439676	**1.000,D**	C/C
1362	ZNF493	p.C323F	rs4621113	**1.000,D**	G/T
1363	PKD1L2	p.G1163W	rs4889244	**1.000,D**	T/T
1364	OR2L8	p.Y217C	rs4925583	**1.000,D**	G/G
1365	SLC22A24	p.R172G	rs4963245	**1.000,D**	G/C
1366	OR5AU1	p.L117F	rs4982419	**1.000,D**	A/A
1367	OR1D5	p.L250F	rs55677131	**1.000,D**	G/A
1368	OR51A2	p.S218F	rs55889636	**1.000,D**	A/A
1369	SPINT4	p.G73S	rs6017667	**1.000,D**	G/A
1370	MRC1	p.G396S	rs606231248	**1.000,D**	G/A
1371	GLT6D1	p.A216T	rs61739510	**1.000,D**	C/T
1372	TPSD1	p.L70F	rs61739908	**1.000,D**	C/T
1373	ARRDC4	p.T235M	rs61747226	**1.000,D**	C/T
1374	OR5A1	p.D183N	rs6591536	**1.000,D**	A/A
1375	POU5F1B	p.E238Q	rs7002225	**1.000,D**	G/C
1376	KCNJ12	p.D173N	rs73313922	**1.000,D**	G/A
1377	CCDC157	p.D51N	rs740223	**1.000,D**	G/A
1378	KCNJ12	p.G145S	rs75029097	**1.000,D**	G/A
1379	KCNJ12	p.E139K	rs76265595	**1.000,D**	G/A
1380	GOLGA3	p.G608C	rs768043775	**1.000,D**	C/A
1381	EPHA10	p.G187R	rs912259721	**1.000,D**	C/G
**opGain SNP**					
1382	AADACL3	p.C13X	rs3000860	N/A	C/A
1383	TTC22	p.R342X	rs2270002	N/A	G/A

1384	NBPF8	p.Y905X	rs878920914	N/A	C/G
1385	FCGR2A	p.Q62X	rs9427397	N/A	C/T
1386	FMO2	p.Q472X	rs6661174	N/A	T/T
1387	TLR5	p.R392X	rs5744168	N/A	G/A
1388	OR2L8	p.Y289X	rs10888281	N/A	A/A
1389	ANKRD36C	p.R417X	rs76474100	N/A	G/A
1390	DBI	p.Y28X	rs3731608	N/A	C/G
1391	TEX51	p.R72X	rs61730220	N/A	C/T
1392	LOC100130451	p.S82X	rs13000409	N/A	C/C
1393	C2orf83	p.W141X	rs2176186	N/A	C/T
1394	TTLL3	p.R704X	rs115917139	N/A	C/T
1395	LOC100132146	p.R46X	rs148870990	N/A	C/T
1396	CPN2	p.Q509X	rs4974539	N/A	G/A
1397	ADH4	p.Y22X	rs3919370	N/A	A/T
1398	SLC6A18	p.Y319X	rs7447815	N/A	C/G
1399	ADAMTS12	p.L230X	rs1530507	N/A	T/T
1400	ANKDD1B	p.W480X	rs34358	N/A	G/A
1401	FAM81B	p.Q144X	rs35391433	N/A	C/T
1402	DCANP1	p.R117X	rs12520799	N/A	T/A
1403	SRA1	p.S93X	rs250426	N/A	G/T
1404	OR2J1	p.Q194X	rs2394517	N/A	C/T
1405	CCHCR1	p.E41X	rs72856718	N/A	C/A
1406	HLA-DRB1	p.W38X	rs9269958	N/A	C/T
1407	HLA-DPB1	p.G40X	rs1126511	N/A	G/T
1408	TAAR9	p.K61X	rs2842899	N/A	T/T
1409	AMZ1	p.R292X	rs55919423	N/A	C/T
1410	VWDE	p.R230X	rs17165936	N/A	G/A
1411	DNAH11	p.E34X	rs2285943	N/A	T/T
1412	ZNF117	p.R428X	rs1404453	N/A	A/A
1413	TYW1B	p.W373X	rs3015858	N/A	T/T
1414	LRRD1	p.E329X	rs200099667	N/A	C/A
1415	PVRIG	p.E18X	rs7786505	N/A	G/T
1416	MUC3A	p.Q2944X	rs79874934	N/A	C/T
1417	PRSS1	p.G177X	rs1240508430	N/A	G/T
1418	LPL	p.S474X	rs328	N/A	C/G
1419	IDO2	p.Y359X	rs4503083	N/A	T/A
1420	VPS13B	p.Y413X	rs7460625	N/A	G/G
1421	IFNE	p.Q71X	rs2039381	N/A	G/A
1422	KIAA1161	p.Y23X	rs4879782	N/A	G/C
1423	OR1B1	p.R192X	rs1476860	N/A	G/A
1424	POMT1	p.Q251X	rs3887873	N/A	C/T
1425	ARMS2	p.R38X	rs2736911	N/A	C/T
1426	PSTK	p.C181X	rs1848991631	N/A	T/A
1427	OR51Q1	p.R236X	rs2647574	N/A	C/T
1428	OR4X1	p.Y273X	rs10838851	N/A	A/A

1429	OR4C16	p.Q17X	rs1459101	N/A	C/T
1430	OR5AR1	p.Q19X	rs11228710	N/A	C/T
1431	SLC22A24	p.Y501X	rs11231341	N/A	C/C
1432	SLC22A10	p.W96X	rs1790218	N/A	A/A
1433	ACTN3	p.R620X	rs1815739	N/A	C/T
1434	CEP295	p.L2557X	rs980660332	N/A	T/G
1435	OR8G1	p.Y259X	rs4268525	N/A	G/G
1436	TAS2R31	p.W300X	rs78820764	N/A	C/T
1437	TAS2R46	p.W250X	rs2708381	N/A	C/T
1438	PRB4	p.R39X	rs12829245	N/A	G/A
1439	SPERT	p.C71X	rs80072371	N/A	C/A
1440	GPR33	p.R140X	rs17097921	N/A	A/A
1441	GOLGA6L6	p.E474X	rs199807662	N/A	C/A
1442	SPATA8	p.R34X	rs3812907	N/A	C/T
1443	NTHL1	p.Q90X	rs150766139	N/A	G/A
1444	PKD1L3	p.R789X	rs4788587	N/A	G/A
1445	PKD1L2	p.Q220X	rs7499011	N/A	G/A
1446	GGT6	p.R152X	rs7215121	N/A	G/A
1447	C17orf107	p.W152X	rs35400274	N/A	G/A
1448	MAP2K3	p.Q73X	rs55796947	N/A	C/T
1449	KRT37	p.Q235X	rs78158550	N/A	G/A
1450	KRT38	p.Q235X	rs148768443	N/A	G/A
1451	C17orf77	p.C207X	rs545652	N/A	C/A
1452	SERPINB11	p.E90X	rs4940595	N/A	G/T
1453	PSG7	p.R98X	rs113247044	N/A	G/A
1454	ZNF283	p.Y599X	rs756423061	N/A	T/G
1455	FUT2	p.W154X	rs601338	N/A	A/A
1456	DHDH	p.Q233X	rs10423255	N/A	C/T
1457	LILRA2	p.W175X	rs1455280111	N/A	G/A
1458	LILRA1	p.G261X	rs150508449	N/A	G/T
1459	USP29	p.Y913X	rs9973206	N/A	A/A
1460	LOC388780	p.Q52X	rs6137326	N/A	C/T
1461	SIGLEC1	p.E88X	rs150358287	N/A	C/A
1462	KIAA1755	p.R510X	rs41282820	N/A	G/A
1463	TPTE	p.R229X	rs1810540	N/A	C/T
1464	KRTAP13-2	p.C135X	rs877346	N/A	A/T
1465	KRTAP10-6	p.C261X	rs233303	N/A	G/T
1466	GAB4	p.G163X	rs28502153	N/A	C/A
1467	CLDN5	p.Q37X	rs885985	N/A	G/A
1468	RFPL1	p.Q243X	rs12484086	N/A	T/T
1469	MAGEB16	p.R272X	rs4829392	N/A	T/T
1470	OR2T35	p.I320_R321delinsX	rs370874670	N/A	///
1471	NRP2	p.C901_X902delinsX	rs200483574	N/A	///
1472	CCDC66	p.L3_G4delinsLGX	rs150150392	N/A	///
1473	RETNLB	p.L14_L15delinsX	rs368497660	N/A	///

1474	HLA-DQA1	p.M99X	rs199556640	N/A	///
1475	FAM20C	.L317_D318delinsLDR)	rs771282640	N/A	///
1476	VWDE	p.K1273_E1274delinsX	rs11454536	N/A	///
1477	TYW1B	p.E339_S340delinsX	rs11354897	N/A	///
1478	SSPO	p.Y3861_C3862delinsX	N/A	N/A	///
1479	KMT2C	p.Y816_I817delinsX	rs150073007	N/A	///
1480	LILRB2	p.L241X	rs2080410040	N/A	///
1481	OR12D1	N/A	rs28993580	N/A	///
1482	PKD1L2	N/A	rs145602984	N/A	///
1483	MROH8	p.A37delinsPCLX	rs11467214	N/A	///
1484	MROH8	p.Lys31X	rs57415986	N/A	///
nDel Exonic					
1485	FAM231B	p.F71fs	rs57681900	frameshift deletion	
1486	MST1L	p.G298fs	rs59375146	frameshift deletion	
1487	MST1L	p.Q40fs	rs113982165	frameshift deletion	
1488	UBXN11	p.P499fs	rs761408866	frameshift deletion	
1489	UBXN11	p.G496fs	rs749678087	frameshift deletion	
1490	AIM1L	p.P538fs	rs759140804	frameshift deletion	
1491	AIM1L	p.G536fs	rs755623773	frameshift deletion	
1492	AIM1L	p.L524fs	rs1460539649	frameshift deletion	
1493	CYP4B1	p.D295fs	rs3215983	frameshift deletion	
1494	NBPF8	p.K39fs	N/A	frameshift deletion	
1495	HRNR	p.M1fs	rs34061715	frameshift deletion	
1496	KIAA0040	p.K68fs	rs754204844	frameshift deletion	
1497	KIAA0040	p.N65fs	rs762930078	frameshift deletion	
1498	ACTN2	p.P31fs	rs11355106	frameshift deletion	
1499	OR2B11	p.F8fs	rs35305980	frameshift deletion	
1500	OR2T4	p.I253fs	rs34079073	frameshift deletion	
1501	OR2T35	p.C203fs	rs72452004	frameshift deletion	
1502	KIAA1841	p.G695fs	rs142269591	frameshift deletion	
1503	SULT1C3	p.K222fs	rs149535765	frameshift deletion	
1504	SCRN3	p.I411fs	rs145699077	frameshift deletion	
1505	NRP2	p.S899fs	rs527478913	frameshift deletion	
1506	CCR5	p.Y184fs	rs333	frameshift deletion	
1507	FRG2C	p.R161fs	rs373728386	frameshift deletion	
1508	OR5K4	p.K299fs	rs11288615	frameshift deletion	
1509	POLQ	p.I1421fs	rs546221341	frameshift deletion	
1510	ALG1L2	p.G156fs	rs55800015	frameshift deletion	
1511	COL6A5	p.H2590fs	rs11355796	frameshift deletion	
1512	HTR3E	p.R21fs	rs397897677	frameshift deletion	
1513	CLDN16	p.R55fs	rs368234054	frameshift deletion	
1514	MUC20	p.A344fs	rs1203696921	frameshift deletion	
1515	MUC20	p.V205fs	N/A	frameshift deletion	
1516	MUC20	p.A344fs	rs144288174	frameshift deletion	
1517	MUC4	p.Q3693fs	rs774501141	frameshift deletion	

1518	CRIPAK	p.H201fs	rs557758398	frameshift deletion
1519	SLC9B1	p.V446fs	rs3974499	frameshift deletion
1520	MAML3	p.Q502fs	rs373804063	frameshift deletion
1521	MAML3	p.Q498fs	rs58287721	frameshift deletion
1522	DCHS2	p.N1365fs	rs140019361	frameshift deletion
1523	SPATA4	p.R279fs	rs28381989	frameshift deletion
1524	PCDHA4	p.R71fs	rs543774145	frameshift deletion
1525	PCDHA4	p.G73fs	rs563991668	frameshift deletion
1526	TIGD6	p.Q344fs	rs3832324	frameshift deletion
1527	SPARC	p.*342fs	rs71757813	frameshift deletion
1528	HLA-A	p.Q250fs	rs45576436	frameshift deletion
1529	HLA-B	p.Q94fs	rs750527298	frameshift deletion
1530	HLA-B	p.R68fs	rs200186034	frameshift deletion
1531	MICA	p.G180fs	rs67841474	frameshift deletion
1532	BTNL2	p.H151fs	rs370253771	frameshift deletion
1533	HLA-DRB5	p.A103fs	rs780328684	frameshift deletion
1534	HLA-DRB5	p.Q39fs	rs774167796	frameshift deletion
1535	HLA-DRB1	p.A103fs	rs67476479	frameshift deletion
1536	HLA-DRB1	p.R101fs	rs758506454	frameshift deletion
1537	HLA-DRB1	p.L37fs	rs767010367	frameshift deletion
1538	HLA-DQA1	p.E73fs	rs760671632	frameshift deletion
1539	HLA-DQA1	p.V91fs	rs758294111	frameshift deletion
1540	MYCT1	p.R21fs	rs3841162	frameshift deletion
1541	SLC22A1	p.P425fs	rs113569197	frameshift deletion
1542	TBP	p.Q76fs	rs796868804	frameshift deletion
1543	TBP	p.Q77fs	rs770128377	frameshift deletion
1544	MEOX2	p.H69fs	rs745896587	frameshift deletion
1545	MEOX2	p.H69fs	rs780233864	frameshift deletion
1546	POMZP3	p.L187fs	rs71819724	frameshift deletion
1547	ZAN	p.G2344fs	rs369526619	frameshift deletion
1548	MUC3A	p.A352fs	rs1584799673	frameshift deletion
1549	MUC3A	p.S2935fs	rs764123091	frameshift deletion
1550	ASB15	p.A240fs	rs138215101	frameshift deletion
1551	PRSS2	p.C234fs	rs554627905	frameshift deletion
1552	IQCA1L	p.E459fs	rs1317223808	frameshift deletion
1553	ERI1	p.*350fs	rs140242735	frameshift deletion
1554	PEBP4	p.N220fs	rs35121552	frameshift deletion
1555	FAM205C	p.A119fs	rs151257262	frameshift deletion
1556	CBWD5	p.S102fs	rs1187604683	frameshift deletion
1557	ANKRD20A1	p.L259fs	rs1190757672	frameshift deletion
1558	HSDL2	p.I170fs	rs10538330	frameshift deletion
1559	RABEPK	p.S133fs	rs546948946	frameshift deletion
1560	GLT6D1	p.W143fs	rs34217442	frameshift deletion
1561	CASP7	p.L43fs	rs10553596	frameshift deletion
1562	CHST15	p.R456fs	rs398015013	frameshift deletion

1563	TMEM80	p.T271fs	rs35782494	frameshift deletion
1564	MUC6	p.Y1920fs	rs752117134	frameshift deletion
1565	OR52B4	p.L41fs	rs11310407	frameshift deletion
1566	OR51F1	p.R92fs	rs34672924	frameshift deletion
1567	OR52Z1P	p.G8fs	rs5789371	frameshift deletion
1568	OR51B5	p.A66fs	rs147062602	frameshift deletion
1569	OR8U8,OR8U1	p.I109fs	rs767571255	frameshift deletion
1570	FOLR3	p.Y16fs	rs71891516	frameshift deletion
1571	MYO7A	p.G1172fs	rs111033223	frameshift deletion
1572	AKAP3	p.S700fs	rs67512580	frameshift deletion
1573	TAS2R43	p.G253fs	rs765322779	frameshift deletion
1574	OR6C76	p.K308fs	rs397719965	frameshift deletion
1575	PTGES3	wholegene	rs10579382	frameshift deletion
1576	LRRIQ1	p.E1699fs	rs398102301	frameshift deletion
1577	NOC4L	p.D299fs	rs1565960277	frameshift deletion
1578	NEK3	p.T293fs	rs1277302339	frameshift deletion
1579	IFI27	p.V41fs	rs3064076	frameshift deletion
1580	C14orf180	p.*161fs	rs111285011	frameshift deletion
1581	NIPA2	p.*361fs	rs368460716	frameshift deletion
1582	GOLGA6L2	p.R694fs	rs375981990	frameshift deletion
1583	GOLGA6L2	p.E673fs	rs763821476	frameshift deletion
1584	GOLGA6L2	p.E659fs	rs756870099	frameshift deletion
1585	CHRFAM7A	p.L76fs	rs201490160	frameshift deletion
1586	LOC283710	p.P25fs	rs3835087	frameshift deletion
1587	TPSD1	p.Q42fs	rs3830782	frameshift deletion
1588	UNKL	p.R245fs	rs1567228821	frameshift deletion
1589	CNOT1	p.F1543fs	rs5817153	frameshift deletion
1590	PKD1L2	p.N236fs	rs548490632	frameshift deletion
1591	ZFPM1	p.E444fs	rs796900280	frameshift deletion
1592	ZFPM1	p.P445fs	rs67322929	frameshift deletion
1593	CTU2	p.R437fs	rs11278302	frameshift deletion
1594	GP1BA	p.T435fs	rs758041497	frameshift deletion
1595	GP1BA	p.S441fs	rs770089708	frameshift deletion
1596	KRTAP29-1	p.P173fs	rs144150438	frameshift deletion
1597	EFCAB13	p.S269fs	rs10538163	frameshift deletion
1598	CCDC40	p.T1027fs	rs71163918	frameshift deletion
1599	TGIF1	p.P82fs	rs11571510	frameshift deletion
1600	ANKLE1	p.G630fs	rs58535756	frameshift deletion
1601	ZNF626	p.A461fs	N/A	frameshift deletion
1602	FCGBP	p.E1221fs	N/A	frameshift deletion
1603	FCGBP	p.R1196fs	N/A	frameshift deletion
1604	FCGBP	p.Q1188fs	rs1374568639	frameshift deletion
1605	FCGBP	p.V1184fs	rs1213861174	frameshift deletion
1606	ZNF283	wholegene	rs71907168	frameshift deletion
1607	DHDH	p.E294fs	rs3835153	frameshift deletion

1608	ZNF480	p.L2fs	rs3217319	frameshift deletion
1609	ZNF880	p.K105fs	rs34470614	frameshift deletion
1610	LILRB5	p.P599fs	N/A	frameshift deletion
1611	LILRB1	p.P366fs	rs2064059683	frameshift deletion
1612	LILRB1	p.Y373fs	rs2064062450	frameshift deletion
1613	2DL1,KIR2DS5,KIR2	p.K176fs	rs767604061	frameshift deletion
1614	KIR2DL4	p.M318fs	rs1280409609	frameshift deletion
1615	ZNF211	p.V104fs	rs34418076	frameshift deletion
1616	ZNF544	p.R93fs	rs570026104	frameshift deletion
1617	DEFB126	p.G53fs	rs11467497	frameshift deletion
1618	DEFB126	p.T105fs	rs11467417	frameshift deletion
1619	KRTAP19-6	p.F57fs	rs5843453	frameshift deletion
1620	SON	p.R2416fs	rs34373121	frameshift deletion
1621	KRTAP10-1	p.D159fs	rs782130783	frameshift deletion
1622	COL18A1	p.G942fs	rs149296338	frameshift deletion
1623	DDTL	p.G114fs	rs199896117	frameshift deletion
1624	APOL4	p.E108fs	N/A	frameshift deletion
1625	NUDT11	wholegene	rs782744505	frameshift deletion
1626	SLC25A5	p.A150fs	rs759019641	frameshift deletion
1627	MUC19	N/A	rs1163167564	frameshift deletion
1628	MUC19	N/A	rs1308102439	frameshift deletion
1629	MUC19	N/A	rs112524759	frameshift deletion
1630	MUC19	N/A	N/A	UNKNOWN deletion
1631	CDCP2	p.M409fs	rs3841798	frameshift insertion
1632	CDCP2	p.P408fs	rs36013100	frameshift insertion
1633	RHBG	p.S392fs	rs587735548	frameshift insertion
1634	GPATCH4	p.F357fs	rs10637036	frameshift insertion
1635	FMO2	p.A527fs	rs2234889	frameshift insertion
1636	OR2W3	p.V297fs	rs112696640	frameshift insertion
1637	PMS1	p.F159fs	rs3214425	frameshift insertion
1638	OR5K3	p.M299fs	rs144759043	frameshift insertion
1639	ATG3	p.L307fs	rs35560667	frameshift insertion
1640	CRIPAK	p.R154fs	rs759566743	frameshift insertion
1641	CPEB2	p.G486fs	N/A	frameshift insertion
1642	SRA1	p.V110fs	rs3085220	frameshift insertion
1643	SRA1	p.V110fs	rs5871740	frameshift insertion
1644	ABCF1	p.X846delinsX	rs4148252	frameshift insertion
1645	PSORS1C1	p.R37fs	rs138474986	frameshift insertion
1646	HLA-B	p.Q94fs	rs749557905	frameshift insertion
1647	HLA-B	p.E69fs	rs9281379	frameshift insertion
1648	HLA-B	p.E69fs	rs9281379	frameshift insertion
1649	MICA	p.G180fs	rs41293539	frameshift insertion
1650	HLA-DRB5	p.D99fs	rs771796054	frameshift insertion
1651	HLA-DRB5	p.E98fs	rs776055221	frameshift insertion
1652	HLA-DRB5	p.Q39fs	rs766955366	frameshift insertion

1653	HLA-DRB5	p.L15fs	rs756922265	frameshift insertion
1654	HLA-DRB5	p.L15fs	rs745343465	frameshift insertion
1655	HLA-DRB5	p.L6fs	rs747906579	frameshift insertion
1656	HLA-DRB1	p.A100fs	rs200320734	frameshift insertion
1657	HLA-DRB1	p.P40fs	rs749085224	frameshift insertion
1658	HLA-DQA1	p.F74fs	rs764189141	frameshift insertion
1659	HLA-DQA1	p.A92fs	rs777760029	frameshift insertion
1660	HLA-DQA1	p.I98fs	rs9282026	frameshift insertion
1661	CCDC129	p.P1032fs	rs35589779	frameshift insertion
1662	ZAN	p.C1923fs	N/A	frameshift insertion
1663	MUC3A	p.I351fs	rs1385837141	frameshift insertion
1664	MUC3A	p.R2936fs	rs756000364	frameshift insertion
1665	ARHGEF5	p.M1fs	rs766185415	frameshift insertion
1666	SSPO	p.S3069fs	rs397815440	frameshift insertion
1667	AGAP3	p.G24fs	rs539642617	frameshift insertion
1668	AGAP3	p.A27fs	rs552859803	frameshift insertion
1669	AGAP3	p.C32fs	rs766984834	frameshift insertion
1670	LAPTM4B	p.R67fs	rs764278197	frameshift insertion
1671	MROH6	p.G676fs	rs551319836	frameshift insertion
1672	IFNK	p.K10fs	rs144060127	frameshift insertion
1673	CBWD3	p.Q257fs	rs200498038	frameshift insertion
1674	OR1J2	p.Y102fs	rs145911830	frameshift insertion
1675	OBP2A	p.Q194fs	rs112851559	frameshift insertion
1676	PTCHD3	p.G308fs	rs112067123	frameshift insertion
1677	TMEM254	p.S13fs	rs113172526	frameshift insertion
1678	MUC6	p.T1922fs	rs754561621	frameshift insertion
1679	C11orf40	p.M201fs	rs141600462	frameshift insertion
1680	C11orf40	p.C200fs	rs67037861	frameshift insertion
1681	OR51B5	p.S187fs	rs113047337	frameshift insertion
1682	OR51I2	p.L238fs	rs35301588	frameshift insertion
1683	OR8J1	p.X317delinsX	rs34649252	frameshift insertion
1684	WNK1	p.L740fs	rs11441897	frameshift insertion
1685	AKAP3	p.G701fs	rs200178782	frameshift insertion
1686	CLECL1	p.S52fs	rs113222621	frameshift insertion
1687	TAS2R43	p.S254fs	rs760672236	frameshift insertion
1688	SLAIN1	p.A73fs	rs201380414	frameshift insertion
1689	SLAIN1	p.Q76fs	rs542815284	frameshift insertion
1690	OR11G2	p.C227fs	rs398077614	frameshift insertion
1691	ZFYVE19	p.W38fs	rs142730574	frameshift insertion
1692	C15orf40	p.L132fs	rs534261421	frameshift insertion
1693	MESP1	p.A53fs	rs759335947	frameshift insertion
1694	MESP1	p.A53fs	rs763577487	frameshift insertion
1695	PGPEP1L	p.V111fs	rs398043625	frameshift insertion
1696	IL32	p.R113fs	rs71818662	frameshift insertion
1697	ZNF778	p.E110fs	rs10625512	frameshift insertion

1698	KRTAP4-8	p.M1fs	rs201764113	frameshift insertion
1699	LOC100134391	p.S283fs	rs58614631	frameshift insertion
1700	TSPAN10	p.F265fs	N/A	frameshift insertion
1701	SETBP1	p.L227fs	rs3085861	frameshift insertion
1702	OR7G3	p.S310fs	rs111279560	frameshift insertion
1703	DCAF15	p.E480fs	rs141180609	frameshift insertion
1704	FCGBP	p.G1220fs	rs1330583248	frameshift insertion
1705	FCGBP	p.D1195fs	N/A	frameshift insertion
1706	FCGBP	p.G1187fs	rs1266581324	frameshift insertion
1707	FCGBP	p.V1186fs	N/A	frameshift insertion
1708	DHDH	p.A170fs	rs3830420	frameshift insertion
1709	EMC10	p.L231fs	rs200206091	frameshift insertion
1710	VSIG10L	p.A859fs	rs11402251	frameshift insertion
1711	SIGLEC12	p.A66fs	rs368715137	frameshift insertion
1712	LILRB2	p.E239fs	rs2080410693	frameshift insertion
1713	LILRB1	p.W367fs	rs2064060119	frameshift insertion
1714	LILRB1	p.T372fs	rs2064061088	frameshift insertion
1715	2DS1,KIR2DS5,KIR2	p.V177fs	rs750502059	frameshift insertion
1716	KIR2DL4	p.S267fs	rs11371265	frameshift insertion
1717	KIR2DL4	p.G319fs	rs1168791468	frameshift insertion
1718	KIR2DS4	p.S151fs	rs551456772	frameshift insertion
1719	GNRH2	p.E101fs	rs16996832	frameshift insertion
1720	KIZ	p.R13fs	rs397791672	frameshift insertion
1721	ADNP	p.K1016fs	rs761350619	frameshift insertion
1722	SON	p.G2412fs	rs34377180	frameshift insertion
1723	PRDM15	p.R88fs	rs202107842	frameshift insertion
1724	KRTAP10-1	p.S160fs	rs781946879	frameshift insertion
1725	TCEAL6	p.Q175fs	rs11408120	frameshift insertion
1726	OR10J4	N/A	rs11419544	frameshift insertion
1727	ABO	N/A	rs8176719	frameshift insertion
1728	MUC19	N/A	rs5797672	frameshift insertion
1729	MUC19	N/A	rs60568788	frameshift insertion
1730	CYP2D6	N/A	rs149012039	frameshift insertion
1731	MUC19	N/A	rs879792835	frameshift insertion
1732	SERPINA2	N/A	N/A	UNKNOWN insertion
1733	CDK11A	p.301_304del	rs199942750	nonframeshift deletion
1734	TMEM52	p.23_26del	rs575852588	nonframeshift deletion
1735	UBXN11	p.490_492del	rs140364749	nonframeshift deletion
1736	NRDC	p.154_155del	rs35723519	nonframeshift deletion
1737	CLCA4	p.877_878del	rs4001061	nonframeshift deletion
1738	GPSM2	p.523_524del	rs35029887	nonframeshift deletion
1739	EFNA3	p.70_73del	rs199552063	nonframeshift deletion
1740	PRG4	p.473_480del	rs770844232	nonframeshift deletion
1741	SYT14	p.186_186del	rs2307890	nonframeshift deletion
1742	ITPKB	p.92_95del	rs147889095	nonframeshift deletion

1743	FMN2	p.54_54del	rs140531536	nonframeshift deletion
1744	FMN2	p.938_949del	rs562038978	nonframeshift deletion
1745	OR14A16	p.163_164del	rs35979231	nonframeshift deletion
1746	OR2T27	p.77_81del	rs368069888	nonframeshift deletion
1747	MYT1L	p.162_163del	rs764655663	nonframeshift deletion
1748	ALLC	p.301_301del	rs201406139	nonframeshift deletion
1749	APOB	p.12_15del	rs17240441	nonframeshift deletion
1750	TSPYL6	p.194_195del	rs76397255	nonframeshift deletion
1751	CEP68	p.139_140del	rs147392467	nonframeshift deletion
1752	APLF	p.312_313del	rs139666972	nonframeshift deletion
1753	AAK1	p.541_542del	rs3832159	nonframeshift deletion
1754	ALMS1	p.12_14del	rs61156725	nonframeshift deletion
1755	ALMS1	p.524_525del	rs797045227	nonframeshift deletion
1756	TCF7L1	p.8_8del	rs566806913	nonframeshift deletion
1757	EIF2AK3	p.21_21del	rs72416210	nonframeshift deletion
1758	ADRA2B	p.301_304del	N/A	nonframeshift deletion
1759	TMEM87B	p.166_167del	rs71385858	nonframeshift deletion
1760	TNFAIP6	p.X278X	rs35060021	nonframeshift deletion
1761	SPATA3	p.29_37del	rs72362780	nonframeshift deletion
1762	GIGYF2	p.1210_1211del	rs10555297	nonframeshift deletion
1763	KIF1A	p.917_918del	rs10594016	nonframeshift deletion
1764	TMIE	p.123_123del	rs10578999	nonframeshift deletion
1765	GPX1	p.11_13del	rs17838762	nonframeshift deletion
1766	RPL29	p.129_131del	rs368982942	nonframeshift deletion
1767	ZNF717	p.650_652del	rs201263569	nonframeshift deletion
1768	OR5H6	p.120_123del	rs398062605	nonframeshift deletion
1769	BBX	p.397_398del	rs34531902	nonframeshift deletion
1770	USF3	p.1470_1473del	rs112313093	nonframeshift deletion
1771	CFAP100	p.411_412del	rs140223152	nonframeshift deletion
1772	YEATS2	p.804_804del	rs146705467	nonframeshift deletion
1773	MUC4	p.3343_3369del	N/A	nonframeshift deletion
1774	MUC4	p.2937_2940del	rs1728040062	nonframeshift deletion
1775	MUC4	p.2937_2940del	rs756208448	nonframeshift deletion
1776	MUC4	p.2853_2900del	rs1560325547	nonframeshift deletion
1777	MUC4	p.2979_3011del	rs1728722813	nonframeshift deletion
1778	MUC4	p.1535_1551del	rs754456588	nonframeshift deletion
1779	MUC4	p.1433_1449del	rs751980714	nonframeshift deletion
1780	MUC4	p.1327_1343del	rs770878205	nonframeshift deletion
1781	MUC4	p.1104_1151del	rs1560389462	nonframeshift deletion
1782	HTT	p.18_19del	rs374076986	nonframeshift deletion
1783	DSPP	p.1019_1024del	rs759454841	nonframeshift deletion
1784	DSPP	p.1023_1024del	rs750849673	nonframeshift deletion
1785	DSPP	p.1025_1035del	rs1238747995	nonframeshift deletion
1786	DSPP	p.1087_1089del	rs367717407	nonframeshift deletion
1787	MAML3	p.768_768del	rs3051167	nonframeshift deletion

1788	CDKN2AIP	p.241_242del	rs10533201	nonframeshift deletion
1789	MAP3K1	p.941_942del	rs10552703	nonframeshift deletion
1790	FOXD1	p.303_303del	N/A	nonframeshift deletion
1791	GFM2	p.641_643del	rs5868753	nonframeshift deletion
1792	MSH3	p.52_57del	rs201874762	nonframeshift deletion
1793	MSH3	p.66_68del	rs144629981	nonframeshift deletion
1794	CHD1	p.1684_1684del	rs138635992	nonframeshift deletion
1795	HAVCR1	p.158_163del	rs141023871	nonframeshift deletion
1796	WWC1	p.861_862del	rs111457550	nonframeshift deletion
1797	SLC34A1	p.91_97del	rs876661296	nonframeshift deletion
1798	DOK3	p.289_289del	rs138153794	nonframeshift deletion
1799	WRNIP1	p.170_173del	rs535821800	nonframeshift deletion
1800	PRSS16	p.506_510del	rs141138864	nonframeshift deletion
1801	MUC22	p.1469_1472del	rs1324521685	nonframeshift deletion
1802	MUC22	p.1475_1478del	N/A	nonframeshift deletion
1803	MUC22	p.1480_1483del	rs1385650348	nonframeshift deletion
1804	CYP21A2	p.6_6del	rs61338903	nonframeshift deletion
1805	NOTCH4	p.15_16del	rs35795312	nonframeshift deletion
1806	HLA-DRB5	p.101_101del	rs770709838	nonframeshift deletion
1807	HLA-DQA1	p.79_79del	rs534654325	nonframeshift deletion
1808	RRP36	p.8_9del	rs200886831	nonframeshift deletion
1809	TTBK1	p.750_750del	rs113160341	nonframeshift deletion
1810	FNDC1	p.1471_1477del	rs141435210	nonframeshift deletion
1811	MRPL18	p.9_10del	rs58504486	nonframeshift deletion
1812	MAP3K4	p.642_643del	rs113801384	nonframeshift deletion
1813	HGC6.3	p.52_80del	rs1798203599	nonframeshift deletion
1814	TBP	p.72_72del	rs71815788	nonframeshift deletion
1815	MEOX2	p.76_77del	rs113582077	nonframeshift deletion
1816	SP8	p.147_147del	rs372591893	nonframeshift deletion
1817	COA1	p.58_58del	rs143305109	nonframeshift deletion
1818	NACAD	p.614_654del	rs1562927994	nonframeshift deletion
1819	NACAD	p.601_641del	rs1562928025	nonframeshift deletion
1820	COBL	p.538_539del	rs142060269	nonframeshift deletion
1821	ZAN	p.1922_1922del	rs1186793581	nonframeshift deletion
1822	MUC3A	p.389_390del	N/A	nonframeshift deletion
1823	LRRC17	p.214_214del	rs3832497	nonframeshift deletion
1824	PODXL	p.28_30del	rs11277659	nonframeshift deletion
1825	CREB3L2	p.100_101del	rs3217268	nonframeshift deletion
1826	KIAA1549	p.54_54del	N/A	nonframeshift deletion
1827	EPHB6	p.165_166del	rs764828501	nonframeshift deletion
1828	OR2A14	p.175_176del	rs34602346	nonframeshift deletion
1829	AGAP3	p.31_32del	rs776100575	nonframeshift deletion
1830	WDR60	p.104_105del	rs145233696	nonframeshift deletion
1831	SGK223	p.1168_1170del	N/A	nonframeshift deletion
1832	SGK223	p.351_352del	N/A	nonframeshift deletion

1833	FDFT1	p.65_66del	rs71711801	nonframeshift deletion
1834	SLC39A14	p.12_14del	rs111662782	nonframeshift deletion
1835	SFRP1	p.13_14del	rs3055861	nonframeshift deletion
1836	RPL7	p.9_9del	rs556746526	nonframeshift deletion
1837	ZFHX4	p.2048_2048del	rs755670320	nonframeshift deletion
1838	MAFA	p.207_208del	rs141816879	nonframeshift deletion
1839	ARHGAP39	p.376_376del	rs138155129	nonframeshift deletion
1840	PRSS3	p.45_45del	rs143332708	nonframeshift deletion
1841	SPAG8	p.182_186del	rs141090907	nonframeshift deletion
1842	MAMDC2	p.355_355del	rs61609258	nonframeshift deletion
1843	PRUNE2	p.2713_2718del	rs11267615	nonframeshift deletion
1844	ZCCHC6	p.226_227del	rs397759922	nonframeshift deletion
1845	NUTM2F	p.691_691del	rs150455117	nonframeshift deletion
1846	MEGF9	p.30_32del	rs369989873	nonframeshift deletion
1847	USP20	p.358_358del	rs10602985	nonframeshift deletion
1848	HMCN2	p.16_18del	rs140466453	nonframeshift deletion
1849	CACNA1B	p.992_997del	rs145816559	nonframeshift deletion
1850	KAT6B	p.1097_1097del	rs71929101	nonframeshift deletion
1851	GSTO1	p.154_154del	rs113200905	nonframeshift deletion
1852	KRTAP5-1	p.110_120del	rs775990209	nonframeshift deletion
1853	KRTAP5-2	p.93_103del	rs59506446	nonframeshift deletion
1854	KRTAP5-5	p.182_191del	rs576867883	nonframeshift deletion
1855	SMPD1	p.35_38del	rs550365194	nonframeshift deletion
1856	SCUBE2	p.22_23del	rs142900716	nonframeshift deletion
1857	NUCB2	p.401_402del	rs3842269	nonframeshift deletion
1858	FNBP4	p.58_59del	rs397711020	nonframeshift deletion
1859	OR4X1	p.190_191del	rs57294041	nonframeshift deletion
1860	MAML2	p.604_607del	rs141671766	nonframeshift deletion
1861	TMPRSS13	p.83_88del	rs201746372	nonframeshift deletion
1862	ADAMTS8	p.21_22del	rs10548872	nonframeshift deletion
1863	ZNF384	p.514_515del	rs3835029	nonframeshift deletion
1864	ATN1	p.488_488del	N/A	nonframeshift deletion
1865	PLBD1	p.25_26del	rs147342083	nonframeshift deletion
1866	PIK3C2G	p.128_129del	rs35277916	nonframeshift deletion
1867	ATG101	p.81_84del	rs143921343	nonframeshift deletion
1868	THAP2	p.102_103del	rs140783391	nonframeshift deletion
1869	PHLDA1	p.194_195del	rs111754051	nonframeshift deletion
1870	FAM109A	p.146_148del	rs139032867	nonframeshift deletion
1871	VSIG10	p.473_474del	rs67582641	nonframeshift deletion
1872	CHFR	p.382_384del	N/A	nonframeshift deletion
1873	ERICH6B	p.135_141del	rs142875900	nonframeshift deletion
1874	ZIC5	p.420_421del	rs71114653	nonframeshift deletion
1875	HOMEZ	p.536_537del	rs35076736	nonframeshift deletion
1876	FBXO33	p.34_37del	rs535061628	nonframeshift deletion
1877	CCDC177	p.177_179del	rs147747237	nonframeshift deletion

1878	CCDC177	p.178_179del	rs375620132	nonframeshift deletion
1879	IRF2BPL	p.123_125del	rs200317113	nonframeshift deletion
1880	RIN3	p.967_967del	rs570458246	nonframeshift deletion
1881	TMEM121	p.293_294del	N/A	nonframeshift deletion
1882	GOLGA6L6	p.577_578del	rs201222558	nonframeshift deletion
1883	GOLGA6L2	p.624_625del	rs796716630	nonframeshift deletion
1884	GOLGA6L2	p.598_599del	rs766696615	nonframeshift deletion
1885	OTUD7A	p.689_689del	rs199744400	nonframeshift deletion
1886	RYR3	p.4438_4439del	rs3217346	nonframeshift deletion
1887	LRRC49	p.342_342del	rs3834543	nonframeshift deletion
1888	GOLGA6L9	p.209_216del	N/A	nonframeshift deletion
1889	WDR73	p.315_321del	rs11267906	nonframeshift deletion
1890	MESP2	p.178_186del	rs200021459	nonframeshift deletion
1891	MESP2	p.183_186del	rs56192595	nonframeshift deletion
1892	FAM174B	p.69_71del	rs66488707	nonframeshift deletion
1893	ATF7IP2	p.60_61del	rs113642662	nonframeshift deletion
1894	TNRC6A	p.111_115del	rs71156436	nonframeshift deletion
1895	IST1	p.243_244del	rs372825060	nonframeshift deletion
1896	ZFPM1	p.446_447del	rs67873604	nonframeshift deletion
1897	PIEZO1	p.1878_1878del	rs150376294	nonframeshift deletion
1898	MPRIP	p.178_179del	rs113250356	nonframeshift deletion
1899	KCNJ12	p.333_333del	rs112163749	nonframeshift deletion
1900	KRT10	p.490_493del	rs778613907	nonframeshift deletion
1901	KRTAP4-1	p.82_85del	rs71155126	nonframeshift deletion
1902	KRTAP9-2	p.139_143del	rs542786200	nonframeshift deletion
1903	KRTAP17-1	p.46_50del	rs796251471	nonframeshift deletion
1904	QRICH2	p.630_639del	rs758349471	nonframeshift deletion
1905	QRICH2	p.581_582del	rs34007000	nonframeshift deletion
1906	USP36	p.958_960del	rs71964596	nonframeshift deletion
1907	SLC38A10	p.1071_1072del	rs10569617	nonframeshift deletion
1908	TXNDC2	p.238_252del	rs781380178	nonframeshift deletion
1909	CABLES1	p.97_99del	rs139352344	nonframeshift deletion
1910	C18orf25	p.313_314del	rs34068795	nonframeshift deletion
1911	POLI	p.14_15del	rs10584411	nonframeshift deletion
1912	ALPK2	p.1006_1011del	rs67925233	nonframeshift deletion
1913	PLIN4	p.731_797del	rs66701788	nonframeshift deletion
1914	KANK3	p.674_675del	rs111905975	nonframeshift deletion
1915	CACNA1A	p.2320_2325del	rs765169827	nonframeshift deletion
1916	URI1	p.298_299del	rs3840928	nonframeshift deletion
1917	DMKN	p.270_271del	rs72334573	nonframeshift deletion
1918	FCGBP	p.2632_2633del	rs1346321490	nonframeshift deletion
1919	NUMBL	p.435_437del	rs141662737	nonframeshift deletion
1920	ZNF284	p.123_124del	rs139900131	nonframeshift deletion
1921	CD3EAP	p.422_422del	rs35729377	nonframeshift deletion
1922	TPRX1	p.206_210del	rs781226540	nonframeshift deletion

1923	ZNF83	p.267_295del	rs1568523939	nonframeshift deletion
1924	FAM71E2	p.778_786del	rs67168196	nonframeshift deletion
1925	DEFB132	p.6_8del	rs371825938	nonframeshift deletion
1926	NINL	p.958_959del	rs34410422	nonframeshift deletion
1927	TP53TG5	p.97_97del	rs10546815	nonframeshift deletion
1928	CTSA	p.29_29del	rs397784956	nonframeshift deletion
1929	KRTAP10-2	p.190_194del	rs67692969	nonframeshift deletion
1930	DRICH1	p.126_127del	rs10564183	nonframeshift deletion
1931	CARD10	p.272_273del	rs113275238	nonframeshift deletion
1932	CDC42EP1	p.253_260del	rs200195385	nonframeshift deletion
1933	TRIOBP	p.398_398del	rs55745992	nonframeshift deletion
1934	TRIOBP	p.538_539del	rs36219868	nonframeshift deletion
1935	BAIAP2L2	p.441_455del	rs371997714	nonframeshift deletion
1936	TCF20	p.322_323del	rs552166978	nonframeshift deletion
1937	SUPT20HL1	p.517_518del	rs762156994	nonframeshift deletion
1938	SUPT20HL1	p.520_526del	rs1569203623	nonframeshift deletion
1939	FAM47A	p.518_530del	N/A	nonframeshift deletion
1940	MUC2	p.Thr4174_Ile4196del	rs1858695544	nonframeshift deletion
1941	MUC19	N/A	rs766853816	nonframeshift deletion
1942	NADK	p.G591delinsEG	rs150880809	nonframeshift insertion
1943	RERE	p.R17delinsRDR	rs201665914	nonframeshift insertion
1944	MDS2	p.V93delinsVR	rs3072132	nonframeshift insertion
1945	SERINC2	p.T308delinsTQ	rs3050461	nonframeshift insertion
1946	FOXD2	p.H287delinsHPH	rs3046924	nonframeshift insertion
1947	IGSF3	p.D1041delinsED	rs576658823	nonframeshift insertion
1948	NBPF12	p.Y932delinsYVLD	rs1553888457	nonframeshift insertion
1949	LCE4A	p.S43delinsSSSGGCC	rs11269814	nonframeshift insertion
1950	LOR	p.G21delinsGGG	rs150026164	nonframeshift insertion
1951	LOR	p.Y189delinsYSGGG	rs11272549	nonframeshift insertion
1952	KCNN3	p.P81delinsQQQQP	rs3831942	nonframeshift insertion
1953	KCNN3	P81delinsQQQQQQQ	N/A	nonframeshift insertion
1954	SEC16B	279delinsGPKAPMKFY	rs762937108	nonframeshift insertion
1955	IGFN1	SVNETGYRKDLGVP	N/A	nonframeshift insertion
1956	HADHB	p.M1delinsMT	rs1064793144	nonframeshift insertion
1957	TMEM247	3delinsQRQHEVVMEQ	rs70940616	nonframeshift insertion
1958	LHCGR	p.P19delinsLQP	rs376653903	nonframeshift insertion
1959	TMEM37	p.T70delinsSVP	rs3217464	nonframeshift insertion
1960	DARS	p.R8delinsASR	rs551090724	nonframeshift insertion
1961	PDE11A	p.P672delinsSP	rs3830637	nonframeshift insertion
1962	ZNF804A	p.N696delinsNT	rs3046266	nonframeshift insertion
1963	FAM171B	p.Q43delinsQQ	rs549897920	nonframeshift insertion
1964	CPS1	p.I5delinsIF	rs61509952	nonframeshift insertion
1965	TTLL3	p.L123delinsLD	rs34761997	nonframeshift insertion
1966	EOMES	p.A120delinsGAA	rs3062761	nonframeshift insertion
1967	RPL14	p.T149delinsTAAAAA	rs369485042	nonframeshift insertion

1968	TRAK1	p.T688delinsTE	rs10634555	nonframeshift insertion
1969	LTF	p.R10delinsRR	rs10662431	nonframeshift insertion
1970	RPL29	p.D131delinsAKD	rs71084167	nonframeshift insertion
1971	CCDC66	p.S606delinsSP	rs112267342	nonframeshift insertion
1972	ATXN7	p.R29delinsRQQ	rs1060499845	nonframeshift insertion
1973	LNP1	nsHPRRHSHEDQEFRC	rs71132521	nonframeshift insertion
1974	USF3	p.Q1473delinsQQQ	rs879138126	nonframeshift insertion
1975	MAGEF1	p.D159delinsED	rs112819846	nonframeshift insertion
1976	MUC4	QATPLPVTSTSSVSTC	N/A	nonframeshift insertion
1977	MUC4	SSVSTGDTTPLPVTDA	rs1727440275	nonframeshift insertion
1978	MUC4	delinsLASTGHTTPLPV	rs771345062	nonframeshift insertion
1979	MUC4	delinsPLPVTDTSSAST	rs1553875496	nonframeshift insertion
1980	MUC4	p.S2026delinsSS	rs112774151	nonframeshift insertion
1981	MUC4	TDASSVSTGHATSLPV	rs1731410319	nonframeshift insertion
1982	MUC4	STGHATPLPVTDNSS	rs1732020095	nonframeshift insertion
1983	MUC4	TSTSSASTGHATPLPV	rs1732040728	nonframeshift insertion
1984	MUC4	p.T113delinsTVTQET	rs142781032	nonframeshift insertion
1985	RNF212	S241delinsWLAPAWA	rs138488801	nonframeshift insertion
1986	CPEB2	p.R169delinsRQ	rs576522935	nonframeshift insertion
1987	POU4F2	p.G55delinsGG	rs530695040	nonframeshift insertion
1988	ZSWIM6	p.P18delinsPG	rs565100893	nonframeshift insertion
1989	MCC	p.S22delinsGS	rs35336557	nonframeshift insertion
1990	KCNN2	p.A53delinsAA	rs151038013	nonframeshift insertion
1991	PRDM6	p.T88delinsTSAS	rs199942027	nonframeshift insertion
1992	SOWAHA	p.L3delinsLAAA	rs566990199	nonframeshift insertion
1993	PCDHA4	p.G70delinsGH	rs200172095	nonframeshift insertion
1994	DIAPH1	p.P618delinsPP	rs3075570	nonframeshift insertion
1995	PCDH12	p.R1182delinsSSSR	rs5871792	nonframeshift insertion
1996	ADAMTS2	p.P24delinsLP	rs568040559	nonframeshift insertion
1997	FOXF2	p.G301delinsGG	rs111257067	nonframeshift insertion
1998	ATXN1	p.H226delinsQH	rs765686161	nonframeshift insertion
1999	NOTCH4	p.L16delinsLL	rs546167192	nonframeshift insertion
2000	HLA-DRB5	p.P7delinsLA	rs774116269	nonframeshift insertion
2001	PRICKLE4	p.L287delinsLL	rs140326303	nonframeshift insertion
2002	FAM46A	p.G44delinsGGDFGG	rs754008809	nonframeshift insertion
2003	PRDM13	p.D371delinsDP	rs112674667	nonframeshift insertion
2004	TSPYL1	p.K177delinsVK	rs397735194	nonframeshift insertion
2005	AKAP12	p.I1530delinsIE	rs113116275	nonframeshift insertion
2006	ARID1B	p.A445delinsAP	rs572236007	nonframeshift insertion
2007	FAM120B	06delinsDPEPRQEVPM	rs1265015773	nonframeshift insertion
2008	TMEM184A	p.S393delinsGS	rs112463195	nonframeshift insertion
2009	ANLN	p.T294delinsTS	rs61549495	nonframeshift insertion
2010	PCLO	p.E2927delinsDE	rs10630259	nonframeshift insertion
2011	FZD1	p.Q88delinsQP	rs139480179	nonframeshift insertion
2012	AKAP9	p.K1335delinsKQ	rs10644111	nonframeshift insertion

2013	DLX6	p.Q44delinsQP	rs559903070	nonframeshift insertion
2014	MUC3A	p.P205delinsPS	rs111723000	nonframeshift insertion
2015	MUC3A	p.P2577delinsPT	rs112753297	nonframeshift insertion
2016	MUC12	p.T18delinsTA	rs150485202	nonframeshift insertion
2017	RP1L1	delinsGTKVIEGLQEER	rs1585963467	nonframeshift insertion
2018	RP1L1	p.T1327delinsET	rs146656804	nonframeshift insertion
2019	BHLHE22	p.S90delinsSG	rs544639534	nonframeshift insertion
2020	BHLHE22	p.G220delinsGSG	rs763704164	nonframeshift insertion
2021	ADCK5	p.L419delinsLGVQGE	rs782071356	nonframeshift insertion
2022	LURAP1L	p.G49delinsGGGG	rs139315731	nonframeshift insertion
2023	PRUNE2	p.S1019delinsLQQSS	rs11267622	nonframeshift insertion
2024	PHF2	p.T987delinsTPASTT	rs149736720	nonframeshift insertion
2025	HABP4	p.E60delinsEAA	rs753920168	nonframeshift insertion
2026	CCDC180	p.E772delinsEEE	rs113264216	nonframeshift insertion
2027	C9orf43	p.R296delinsRQ	rs527300639	nonframeshift insertion
2028	SKIDA1	p.G429delinsEEG	rs112207161	nonframeshift insertion
2029	EBLN1	p.L143delinsLL	rs3059425	nonframeshift insertion
2030	GPRIN2	p.A243delinsEVGA	rs554090811	nonframeshift insertion
2031	KIF20B	p.L1062delinsLKV	rs55921521	nonframeshift insertion
2032	PDZD7	p.S785delinsRSS	rs200896335	nonframeshift insertion
2033	PCGF6	p.P28delinsPPP	rs113359610	nonframeshift insertion
2034	AFAP1L2	p.G111delinsRG	rs60778514	nonframeshift insertion
2035	CTBP2	.A390delinsALQPQPA	rs372118432	nonframeshift insertion
2036	KRTAP5-1	GSKGGCGSCGGCKC	rs1590818751	nonframeshift insertion
2037	KRTAP5-5	p.G43delinsGGCGS	rs71025763	nonframeshift insertion
2038	ART5	p.I25delinsTI	rs3062849	nonframeshift insertion
2039	OR52Z1P	p.I167delinsMGI	rs113029548	nonframeshift insertion
2040	DNHD1	p.A1909delinsAALLH	rs1554888386	nonframeshift insertion
2041	MICALCL	p.A456delinsAPPPP	rs3841216	nonframeshift insertion
2042	SAAL1	p.G11delinsPG	rs148650821	nonframeshift insertion
2043	C11orf80	p.G26delinsGAA	rs567536854	nonframeshift insertion
2044	KRTAP5-7	110delinsSCCQSSCCK	rs771895560	nonframeshift insertion
2045	KRTAP5-8	o.G8delinsGGCGSGCG	rs537752041	nonframeshift insertion
2046	KRTAP5-8	nsSQSSCCKPCCCSSC	rs1565619685	nonframeshift insertion
2047	KRTAP5-10	92delinsGGCGSCGGS	rs71272260	nonframeshift insertion
2048	NOX4	p.M4delinsEM	rs34495256	nonframeshift insertion
2049	DCP1B	p.Q261delinsQQ	rs111543431	nonframeshift insertion
2050	FAM90A1	p.T344delinsTV	rs71265055	nonframeshift insertion
2051	KRT6B	p.L405delinsNL	rs774025074	nonframeshift insertion
2052	KRT2	p.F102delinsGF	rs56850150	nonframeshift insertion
2053	KRT2	p.S101delinsGFGGGS	rs57149265	nonframeshift insertion
2054	KRT4	lelinsAGGFGAGFGTG	rs11267392	nonframeshift insertion
2055	RBM19	p.V296delinsTV	rs55840068	nonframeshift insertion
2056	CAMKK2	p.G539delinsKG	rs398021385	nonframeshift insertion
2057	WDR66	p.G62delinsGEEEEK	rs1030446606	nonframeshift insertion

2058	LRRC43	K333delinsKGEKDKK	rs752145826	nonframeshift insertion
2059	NCOR2	p.G1840delinsSSGG	rs143952466	nonframeshift insertion
2060	NCOR2	p.P511delinsQP	rs372331070	nonframeshift insertion
2061	NCOR2	p.P511delinsQQP	rs35831183	nonframeshift insertion
2062	BRI3BP	p.L15delinsLL	rs546819378	nonframeshift insertion
2063	EP400	p.Q2727delinsQQ	rs60608267	nonframeshift insertion
2064	EP400	p.Q2727delinsQQQ	rs528214697	nonframeshift insertion
2065	RBM23	p.Q394delinsAQ	rs397812315	nonframeshift insertion
2066	ACIN1	p.R644delinsHSR	rs34870944	nonframeshift insertion
2067	NOP9	p.A161delinsAE	rs113258190	nonframeshift insertion
2068	NOP9	p.A161delinsAEE	rs113258190	nonframeshift insertion
2069	DDHD1	p.S113delinsGGS	rs140904345	nonframeshift insertion
2070	ATXN3	delinsQQQQQQQQQ	rs193922928	nonframeshift insertion
2071	RTL1	p.E152delinsEE	rs35401447	nonframeshift insertion
2072	GOLGA6L2	p.A873delinsDA	rs34879341	nonframeshift insertion
2073	GOLGA6L2	G567delinsEAGGEDA	rs748500078	nonframeshift insertion
2074	GOLGA8R	p.D272delinsQD	rs372647671	nonframeshift insertion
2075	ARIH1	p.G78delinsGG	rs1060499844	nonframeshift insertion
2076	LOXL1	p.V260delinsVP	rs776481735	nonframeshift insertion
2077	ARID3B	p.L4delinsLQ	rs770169890	nonframeshift insertion
2078	ASPHD1	p.Q170delinsQG	rs140411458	nonframeshift insertion
2079	JPH3	p.P144delinsPAA	rs71156237	nonframeshift insertion
2080	BCL6B	p.S234delinsSS	rs146207245	nonframeshift insertion
2081	KDM6B	p.L251delinsLPP	rs61462443	nonframeshift insertion
2082	CENPV	p.A22delinsGASA	rs539910742	nonframeshift insertion
2083	FAM83G	p.R820delinsHR	rs3071666	nonframeshift insertion
2084	KRTAP4-5	p.Q82delinsRPSCCQ	rs535144703	nonframeshift insertion
2085	KRTAP9-1	C153delinsCCGSSCCQ	rs71155128	nonframeshift insertion
2086	KRTAP9-9	p.P11delinsPTCCRT	rs540633489	nonframeshift insertion
2087	HOXB1	p.P28delinsHSAP	rs145570960	nonframeshift insertion
2088	EME1	p.K137delinsKQ	rs3060668	nonframeshift insertion
2089	ANKRD40	p.D106delinsDD	rs138572332	nonframeshift insertion
2090	PPM1E	p.P33delinsPEP	rs201186780	nonframeshift insertion
2091	GRIN2C	p.L1049delinsPPEL	rs139495021	nonframeshift insertion
2092	GNAL	p.P36delinsPAL	rs201898548	nonframeshift insertion
2093	GREB1L	p.D1861delinsDL	rs10654340	nonframeshift insertion
2094	RTTN	p.D243delinsGD	rs58913700	nonframeshift insertion
2095	CNDP1	p.V15delinsVL	rs10663835	nonframeshift insertion
2096	KCNG2	p.P8delinsPG	rs71338073	nonframeshift insertion
2097	OR7C2	p.V228delinsVS	rs3044711	nonframeshift insertion
2098	GDF1	p.A157delinsAA	rs571387097	nonframeshift insertion
2099	CEBPA	p.P197delinsHPP	rs762459325	nonframeshift insertion
2100	LSR	p.G518delinsGR	rs112341068	nonframeshift insertion
2101	DMKN	p.G270delinsGSSS	rs763222290	nonframeshift insertion
2102	FCGBP	p.C2635delinsPC	rs1378703310	nonframeshift insertion

2103	FCGBP	p.Q1188delinsLQ	rs1228186040	nonframeshift insertion	
2104	HRC	p.V262delinsDV	rs555002410	nonframeshift insertion	
2105	NR1H2	p.K172delinsKQ	rs34296657	nonframeshift insertion	
2106	ZNF880	p.I138delinsIN	rs34678014	nonframeshift insertion	
2107	HSPBP1	p.S31delinsGGGS	rs10701478	nonframeshift insertion	
2108	SSC5D	p.P1325delinsPT	rs150781976	nonframeshift insertion	
2109	ZNF814	p.E297delinsYE	rs10687775	nonframeshift insertion	
2110	RALY	p.A230delinsAS	rs10649600	nonframeshift insertion	
2111	SYNJ1	p.L1406delinsNTL	rs57257560	nonframeshift insertion	
2112	C21orf58	p.H299delinsHH	rs112899928	nonframeshift insertion	
2113	TMEM191B	p.F159delinsFY	rs547549857	nonframeshift insertion	
2114	MED15	p.L210delinsLQ	rs748572492	nonframeshift insertion	
2115	NEFH	p.A646delinsAKSPEKA	rs147489453	nonframeshift insertion	
2116	BAIAP2L2	p.N412delinsTPMN	rs142739979	nonframeshift insertion	
2117	SYNGR1	p.P202delinsPN	rs149306472	nonframeshift insertion	
2118	DCAF8L2	p.E129delinsEE	rs745536197	nonframeshift insertion	
2119	SHROOM4	p.E1138delinsEE	rs143151534	nonframeshift insertion	
2120	SHROOM4	p.Q1128delinsQQQQQ	rs201922875	nonframeshift insertion	
2121	AR	p.L57delinsLQ	rs3032358	nonframeshift insertion	
2122	AR	p.G456delinsGG	rs760580125	nonframeshift insertion	
2123	RBMXL3	R393delinsRGRSPNAH	rs55659078	nonframeshift insertion	
2124	MUC19	N/A	rs145652182	nonframeshift insertion	
2125	MUC19	N/A	rs528940514	nonframeshift insertion	
StopLoss					
2126	PRSS48	p.X329E	rs2407221	N/A	G/G
2127	TAP2	p.X687Q	rs241448	N/A	A/G
2128	NPSR1	p.X391R	rs10275028	N/A	T/C
2129	PTCHD3	p.X768Q	rs2505323	N/A	G/G
2130	SNCG	p.X127C	rs9864	N/A	T/T
2131	OR1S1	p.X326W	rs7103033	N/A	A/G
2132	TAS2R19	p.X300W	rs79475879	N/A	T/C
2133	CEP89	p.X784Q	rs745961	N/A	A/G
2134	ZNF568	p.X636R	rs1667366	N/A	T/C
2135	NLRP8	p.X1049Y	rs306457	N/A	G/C
2136	ZBP1	p.X249L	rs1267733750	N/A	T/A
2137	FTCDNL1	p.X148delinsL	rs761406357	N/A	///

Table S4. Damaging mutations, unique for Index Patient S13

267 damaging mutations according to HVAR scores identified in patient S13, potential contributors to a unique ACE phenotype. These mutations were identified by subtracting damaging mutations common to a control group of 16 patients without the unique ACE phenotype specific for Patient S13 (Table S3) from the damaging mutations found in patient S13 (Table S2). There are two prediction classifications **D** (Probably damaging, score>=0.909), **P** (**possibly** damaging, **0.446**<=score<=**0.908**).

NO. SNP	GeneName	AAChange	ID	PolyPhen-2 (HVAR)	Genotype
1	PPP1R16B	p.K431Q	rs61752055	**0.451,P**	A/C
2	PSG9	p.A212S	rs150423600	**0.457,P**	C/A
3	HLX	p.Q125H	rs62621984	**0.459,P**	A/C
4	IL16	p.P434S	rs4072111	**0.468,P**	C/T
5	CMYA5	p.D190G	rs10942901	**0.469,P**	A/G
6	KIF26B	p.T1302M	rs199742874	**0.475,P**	C/T
7	NOXO1	p.R39H	rs117304081	**0.486,P**	C/T
8	SAMD9	p.V549L	rs10279499	**0.489,P**	C/A
9	C8orf46	p.A79S	rs61736270	**0.497,P**	G/T
10	KRT37	p.P434S	rs17737019	**0.500,P**	G/A
11	SYCP2L	p.N647D	rs3798751	**0.503,P**	A/G
12	ANKRD18A	p.Y750C	rs2799163	**0.515,P**	T/C
13	CDHR3	p.L506F	rs76067797	**0.522,P**	C/T
14	ZFHX4	p.P1273S	rs61729527	**0.531,P**	C/T
15	GOLGA2	p.R686W	rs72756867	**0.533,P**	G/A
16	LILRA6	p.G149R	rs1052966	**0.535,P**	T/T
17	ZNF653	p.E352K	rs74552618	**0.546,P**	C/T
18	FMO2	p.E314G	rs2020863	**0.555,P**	A/G
19	ITIH2	p.L569V	rs7084817	**0.556,P**	C/G
20	SNTG2	p.S200L	rs6751090	**0.560,P**	C/T
21	NCAPG2	p.A132S	N/A	**0.569,P**	C/A
22	DSPP	p.D1074G	rs202210195	**0.583,P**	A/G
23	OSBPL1A	p.S810P	rs35693789	**0.583,P**	A/G
24	CASP12	p.T21I	rs138698464	**0.589,P**	G/A
25	SHBG	p.P185L	rs6258	**0.591,P**	C/T
26	MAFA	p.G347C	rs62521874	**0.615,P**	A/A
27	MRPL28	p.D160E	rs11557302	**0.628,P**	G/C
28	S1PR2	p.V286A	rs117064827	**0.630,P**	A/G
29	RYR3	p.N2016S	rs942844610	**0.636,P**	A/G
30	ZNF254	p.D593	rs17854260	**0.641,P**	A/G
31	DNAJC17	p.V204L	rs117485355	**0.647,P**	C/G
32	C10orf11	p.S153F	rs35349706	**0.652,P**	T/T
33	MOCOS	p.S22R	rs113873219	**0.652,P**	C/A
34	TSEN54	p.I137L	rs11559205	**0.656,P**	A/C
35	TAS2R43	p.L235F	rs3759244	**0.658,P**	A/A

36	LAMA4	p.R545C	rs138153075	**0.659,P**	G/A
37	FXYD4	p.D28H	rs150156235	**0.669,P**	G/C
38	KRT6C	p.G111D	rs394598	**0.672,P**	T/T
39	PTPRB	p.G1934A	rs17226367	**0.683,P**	C/G
40	IGFN1	p.S3069R	rs61743921	**0.686,P**	C/A
41	RANBP3L	p.V471D	rs145254521	**0.692,P**	A/T
42	SH3TC2	p.M1184V	rs142451273	**0.695,P**	T/C
43	VPS13D	p.S3777C	rs149521489	**0.698,P**	C/G
44	NECAB1	p.A271S	rs115555424	**0.700,P**	G/T
45	TNC	p.R322Q	rs145315080	**0.701,P**	C/T
46	PLEKHG5	p.E786K	rs184242303	**0.703,P**	C/T
47	ZNF254	p.K537N	rs12611425	**0.707,P**	G/C
48	GRIN2C	p.L65F	rs78349823	**0.714,P**	G/A
49	MAPKBP1	p.V386I	rs75869993	**0.718,P**	G/A
50	TGM5	p.R81C	rs773076654	**0.720,P**	G/A
51	HK2	p.R373W	rs199992983	**0.728,P**	C/T
52	CDH4	p.G523S	rs142900721	**0.729,P**	G/A
53	TRPA1	p.E179K	rs920829	**0.739,P**	C/T
54	DNAH1	p.V3406I	rs201752275	**0.740,P**	G/A
55	CP	p.T841R	rs56033670	**0.744,P**	G/C
56	OR51I1	p.R302C	rs61736831	**0.745,P**	G/A
57	YY1AP1	p.D231N	rs41264945	**0.747,P**	C/T
58	ZNF234	p.V420I	rs201556875	**0.747,P**	G/A
59	CPN1	p.G178D	rs61751507	**0.764,P**	C/T
60	LILRA1	p.V387L	rs116973751	**0.764,P**	G/T
61	SLCO5A1	p.G594V	rs34698405	**0.767,P**	C/A
62	FHL2	p.T171M	rs727504674	**0.770,P**	G/A
63	OTOP3	p.M1T	rs368367098	**0.775,P**	T/C
64	TRPM8	p.Y251C	rs17868387	**0.781,P**	A/G
65	CORO2B	p.L189Q	rs138122643	**0.781,P**	T/A
66	ZFR2	p.R521C	rs61742027	**0.786,P**	G/A
67	CAMK2N2	p.K47T	rs1332780006	**0.792,P**	T/G
68	GEMIN5	p.R1016C	rs61749643	**0.792,P**	G/A
69	CABLES2	p.V381L	rs61742254	**0.794,P**	C/G
70	EVC	p.D95G	rs41269547	**0.797,P**	A/G
71	KCNMB1	p.A27V	rs993115575	**0.797,P**	G/A
72	C2CD2L	p.S466T	rs1946807141	**0.805,P**	T/A
73	VTA1	p.C38S	rs2232300	**0.812,P**	T/A
74	SLC6A15	p.E684D	rs145111717	**0.816,P**	C/A
75	RET	p.R982C	rs17158558	**0.818,P**	C/T
76	ALMS1	p.S2101L	rs28730854	**0.824,P**	C/T
77	ZAN	p.A2511V	rs76325149	**0.824,P**	C/T
78	IZUMO4	p.Y137F	rs45506200	**0.824,P**	A/T
79	PCDHB2	p.F516L	rs143150465	**0.826,P**	C/A
80	FOXA1	p.E269V	rs757337935	**0.827,P**	T/A

81	OR51B5	p.T78K	rs57273781	**0.828,P**	G/T
82	ODF4	p.R77C	rs73250854	**0.833,P**	C/T
83	CEP131	p.A1020T	rs117616373	**0.834,P**	C/T
84	KRT75	p.M438T	rs764819403	**0.838,P**	A/G
85	GRWD1	p.A64V	rs1971402770	**0.842,P**	C/T
86	SPANXB1	p.D86H	rs1218473051	**0.848,P**	G/C
87	PRR22	p.P248L	rs34572934	**0.852,P**	G/A
88	DOCK8	p.R1238H	rs767874435	**0.858,P**	G/A
89	MLH3	p.R1152C	rs569011240	**0.858,P**	G/A
90	CCDC96	p.Q231H	rs374259671	**0.859,P**	C/G
91	SHROOM1	p.T488N	rs769565321	**0.859,P**	G/T
92	MYH7B	p.L1663F	rs1319423309	**0.864,P**	C/T
93	S100P	p.V14I	rs187031070	**0.867,P**	G/A
94	OSCAR	p.C101W	rs1488511419	**0.870,P**	G/C
95	DHDH	p.G282R	rs3765148	**0.871,P**	G/A
96	ARHGEF4	p.R650C	rs61758709	**0.872,P**	C/T
97	PLEKHH1	p.G677R	rs61534804	**0.872,P**	G/A
98	NLRP3	p.L331V	rs202077909	**0.873,P**	C/G
99	COL6A3	p.E779K	rs146092501	**0.874,P**	C/T
100	TPSD1	p.A127V	rs143993373	**0.876,P**	C/T
101	DUPD1	p.D66N	rs11594934	**0.881,P**	C/T
102	TMEM99	p.L95R	rs1044806	**0.883,P**	T/G
103	AP5B1	p.G170V	rs12362011	**0.885,P**	C/A
104	PNPLA7	p.W899S	rs61747535	**0.887,P**	C/G
105	MTDH	p.A78S	rs17854373	**0.888,P**	G/T
106	PRKAG1	p.T89S	rs1126930	**0.888,P**	G/C
107	OLFML1	p.T53M	rs141351486	**0.893,P**	C/T
108	ARHGAP1	p.G183E	rs775108640	**0.893,P**	C/T
109	CELSR3	p.Q331R	rs1051589441	**0.906,P**	T/C
110	KRT1	p.A454S	rs17678945	**0.910,D**	C/A
111	CPA4	p.A282T	rs570075096	**0.917,D**	G/A
112	KLLN	p.R128G	rs201652303	**0.918,D**	G/C
113	ZNF404	p.R474C	rs76311065	**0.918,D**	G/A
114	VASN	p.G551R	rs367830549	**0.921,D**	G/A
115	NPAS4	p.G676V	rs1371192899	**0.922,D**	G/T
116	INADL	p.E362A	rs1286823	**0.926,D**	A/C
117	FARP1	p.H644Y	rs61730892	**0.937,D**	C/T
118	KAZN	p.E88D	N/A	**0.941,D**	A/C
119	ARFGAP2	p.S364F	N/A	**0.943,D**	G/A
120	USP21	p.G321D	rs17356051	**0.950,D**	G/A
121	RD3	p.G57V	rs767481165	**0.950,D**	C/A
122	EPYC	p.S150C	rs17784152	**0.950,D**	G/C
123	ITGAM	p.R246Q	rs199671976	**0.950,D**	G/A
124	AARS	p.G931S	rs149377346	**0.951,D**	C/T
125	WFS1	p.R456H	rs1801208	**0.953,D**	G/A

126	BPIFA3	p.A41E	rs17124391	**0.956,D**	C/A
127	NFS1	p.R145W	rs148627243	**0.957,D**	G/A
128	PFAS	p.R811W	rs147698247	**0.958,D**	C/T
129	IDI1	p.G12A	rs4880760	**0.959,D**	C/G
130	TSPAN32	p.R164C	rs148601311	**0.959,D**	C/T
131	FFAR3	p.Y249C	rs763450506	**0.959,D**	A/G
132	FAM169A	p.E511K	rs76455982	**0.960,D**	C/T
133	SLX4	p.S1271F	rs3810813	**0.960,D**	G/A
134	EXO1	p.N279S	rs4149909	**0.961,D**	A/G
135	ACSM1	p.A176P	rs61740631	**0.961,D**	C/G
136	HLA-DRB5	p.K41T	rs200581589	**0.962,D**	T/G
137	ACACB	p.D1481V	rs113524436	**0.962,D**	A/T
138	CCDC22	p.R384C	rs143790434	**0.963,D**	T/T
139	C6orf118	p.G271E	rs17852379	**0.964,D**	C/T
140	TRAPPC5	p.R7C	rs753176463	**0.964,D**	C/T
141	FKBP9	p.V314M	rs150348129	**0.966,D**	G/A
142	TRPM1	p.A503V	rs751743414	**0.966,D**	G/A
143	ADAMTSL4	p.R87P	rs199599791	**0.968,D**	G/C
144	ZNF764	p.R52Q	rs61744716	**0.968,D**	C/T
145	HMCN1	p.G1292V	N/A	**0.972,D**	G/T
146	C10orf71	p.D45G	rs151171891	**0.974,D**	A/G
147	KIAA0430	p.Y223H	rs775204838	**0.974,D**	A/G
148	BAG3	p.I94F	rs145393807	**0.975,D**	A/T
149	EPPK1	p.R1066C	rs116925616	**0.977,D**	G/A
150	C1orf106	p.Y333F	rs41313912	**0.978,D**	A/T
151	SCML4	p.R337W	rs56215636	**0.978,D**	G/A
152	PKD1L1	p.E645D	N/A	**0.978,D**	T/A
153	NUP107	p.E394G	rs899872828	**0.978,D**	A/G
154	FSCN2	p.R406P	rs750102960	**0.979,D**	G/C
155	VWDE	p.C1050Y	rs35171886	**0.980,D**	C/T
156	GATAD2A	p.R293H	rs748958687	**0.980,D**	G/A
157	NCAPG2	p.A132V	N/A	**0.981,D**	G/A
158	IPPK	p.R130W	rs75957544	**0.981,D**	G/A
159	PKP1	p.R684W	rs61818256	**0.982,D**	C/T
160	SCFD2	p.G283V	rs79025139	**0.982,D**	C/A
161	TYSND1	p.S239R	rs1166621779	**0.982,D**	T/G
162	MYOM1	p.E704K	rs149528866	**0.983,D**	C/T
163	SLC25A5	p.L173P	rs200606066	**0.983,D**	T/C
164	SVOPL	p.I362R	rs773196860	**0.984,D**	A/C
165	ST8SIA4	p.S221N	rs759794120	**0.986,D**	C/T
166	FAM21A	p.V1285M	rs1427726813	**0.986,D**	G/A
167	EP400	p.P2792S	rs542350095	**0.986,D**	C/T
168	PDPR	p.G564S	rs369641860	**0.986,D**	G/A
169	POLQ	p.A2547V	rs2306211	**0.987,D**	G/A
170	PDCD4	p.M401K	rs201057391	**0.987,D**	T/A

171	MPHOSPH8	p.D460Y	rs75390100	0.988,D	G/T
172	VKORC1	p.D36Y	rs61742245	0.988,D	C/A
173	PMFBP1	p.E193K	rs35370634	0.989,D	C/T
174	FNDC3B	p.A452S	rs35409041	0.990,D	G/T
175	CLDN3	p.P134L	rs139191328	0.990,D	G/A
176	CUBN	p.N2157D	rs144360241	0.990,D	T/C
177	CROCC	p.D463Y	rs114323849	0.991,D	G/T
178	FBXO39	p.L231F	rs1509123	0.991,D	C/T
179	LOXHD1	p.G632C	rs35088381	0.991,D	C/A
180	FAM81B	p.R134C	rs76962324	0.992,D	C/T
181	ZNF703	p.P307S	rs79707182	0.992,D	C/T
182	PLEKHA6	p.A128T	rs61755454	0.993,D	C/T
183	PEX6	p.A809V	rs35830695	0.993,D	G/A
184	FBLN5	p.V361I	rs757825263	0.993,D	C/T
185	MYH13	p.R1438C	rs141961168	0.993,D	G/A
186	FER1L5	p.R1523Q	rs745681758	0.994,D	G/A
187	MRI1	p.T209R	rs141252695	0.994,D	C/G
188	TMF1	p.D798H	rs1532918	0.995,D	C/G
189	DCK	p.P122S	rs67437265	0.995,D	C/T
190	APLF	p.Q433K	rs36021078	0.996,D	C/A
191	TTC21A	p.R574W	rs35581078	0.996,D	C/T
192	TCP10L2	p.G254R	rs200019718	0.996,D	G/A
193	SVEP1	p.E3412Q	rs145193955	0.996,D	C/G
194	OR1N2	p.F194L	rs41297203	0.996,D	T/C
195	A2ML1	p.I1054T	rs371117844	0.996,D	T/C
196	RNF40	p.L261F	rs150097902	0.996,D	C/T
197	CD300C	p.T71I	rs11870245	0.996,D	G/A
198	CATSPERD	p.E596D	rs143660393	0.996,D	G/C
199	MATN3	p.E252K	rs52826764	0.997,D	C/T
200	OGG1	p.N151I	rs764308935	0.997,D	A/T
201	USP19	p.V872A	N/A	0.997,D	A/G
202	FAM65B	p.W15R	rs148823241	0.997,D	A/G
203	HLA-DRB5	p.L28F	rs72508462	0.997,D	C/G
204	OR2AG2	p.G233R	rs143018775	0.997,D	C/T
205	DNAH10	p.T1986M	rs33935373	0.997,D	C/T
206	SLC25A5	p.G183V	rs200974395	0.997,D	G/T
207	OR10X1	p.T90M	rs77690058	0.998,D	G/A
208	UGT2A2,UGT2A1	p.S444Y	rs144056072	0.998,D	G/T
209	IGF2R	p.R292Q	rs370150079	0.998,D	G/A
210	BMS1	p.R939G	rs779750824	0.998,D	C/G
211	TRPM5	p.V335L	rs34350821	0.998,D	C/A
212	OR51I2	p.R263C	rs75620804	0.998,D	C/T
213	TAS2R31	p.D45H	rs143614038	0.998,D	C/G
214	CRAMP1	p.P1084S	rs61746451	0.998,D	C/T
215	FADS6	p.V256I	rs7219093	0.998,D	C/T

216	DOPEY2	p.R2055C	rs145488940	**0.998,D**	C/T
217	HIVEP3	p.P407T	rs41269477	**0.999,D**	G/T
218	IL23R	p.R381Q	rs11209026	**0.999,D**	G/A
219	PVRL4	p.P104T	rs35434391	**0.999,D**	G/T
220	CAPN8	p.A136V	rs71644745	**0.999,D**	G/A
221	TTN	p.Y13023H	rs775496863	**0.999,D**	A/G
222	ESPNL	p.L866Q	N/A	**0.999,D**	T/A
223	SNED1	p.S270Y	rs201554730	**0.999,D**	C/A
224	CDC25C	p.G375R	rs11567997	**0.999,D**	C/G
225	DLGAP2	p.T375M	rs767514905	**0.999,D**	C/T
226	ITGB1	p.I782F	N/A	**0.999,D**	T/A
227	AVPI1	p.L36R	rs11556392	**0.999,D**	A/C
228	OR51B5	p.R88G	rs57900141	**0.999,D**	T/C
229	RTN3	p.D501H	rs7936660	**0.999,D**	G/C
230	KRTAP3-2	p.P53L	rs150830376	**0.999,D**	G/A
231	TSEN54	p.P483A	rs62088470	**0.999,D**	C/G
232	ANKLE1	p.R548Q	rs77683348	**0.999,D**	G/A
233	SYNGR4	p.R27W	rs919804	**0.999,D**	C/T
234	EPHA10	p.G187R	rs912259721	**1.000,D**	C/G
235	ERICH3	p.G319R	rs140864337	**1.000,D**	C/G
236	SCN11A	p.L1158P	rs141686175	**1.000,D**	A/G
237	IGSF10	p.D2614N	rs112889898	**1.000,D**	C/T
238	PDHA2	p.R286P	rs147966234	**1.000,D**	G/C
239	PCDHA1	p.N449H	rs3733712	**1.000,D**	A/C
240	SCML4	p.G158D	rs142985964	**1.000,D**	C/T
241	CFTR	p.R668C	rs1800100	**1.000,D**	C/T
242	FER1L6	p.G943W	rs200894396	**1.000,D**	G/T
243	ACTL7A	p.G214S	rs41278347	**1.000,D**	G/A
244	MUSK	p.A763T	rs199507468	**1.000,D**	G/A
245	FANK1	p.P12L	rs17153879	**1.000,D**	C/T
246	UBASH3B	p.Y546C	rs1428664827	**1.000,D**	A/G
247	GOLGA3	p.G608C	rs768043775	**1.000,D**	C/A
248	GMPR2	p.G260D	rs34354104	**1.000,D**	G/A
249	ARRDC4	p.T235M	rs61747226	**1.000,D**	C/T
250	ATP2C2	p.G809W	rs141229929	**1.000,D**	G/T
251	OR1D2	p.T240I	rs4300683	**1.000,D**	G/A
252	LOXHD1	p.G626C	rs34589386	**1.000,D**	C/A
Stopgain					
253	AMZ1	p.R292X	rs55919423	**1.000,D**	
254	CEP295	p.L2557X	rs980660332	**1.000,D**	
255	DHDH	p.Q233X	rs10423255	**1.000,D**	
256	KIAA1755	p.R510X	rs41282820	**1.000,D**	
257	KRT38	p.Q235X	rs148768443	**1.000,D**	
258	LILRA1	p.G261X	rs150508449	**1.000,D**	
259	LOC100132146	p.R46X	rs148870990	**1.000,D**	

260	LRRD1	p.E329X	rs200099667	**1.000,D**	
261	NTHL1	p.Q90X	rs150766139	**1.000,D**	
262	PSTK	p.C181X	rs1848991631	**1.000,D**	
263	SIGLEC1	p.E88X	rs150358287	**1.000,D**	
264	TEX51	p.R72X	rs61730220	**1.000,D**	
265	TTLL3	p.R704X	rs115917139	**1.000,D**	
266	ZNF283	p.Y599X	rs756423061	**1.000,D**	
Stoploss					
267	ZBP1	p.X249L	rs1267733750	**1.000,D**	
